# Estimates of SARS-CoV-2 Infections and Population Immunity After the COVID-19 Pandemic in Austria: Analysis of National Wastewater Data

**DOI:** 10.1093/infdis/jiaf054

**Published:** 2025-02-18

**Authors:** Uwe Riedmann, Alena Chalupka, Lukas Richter, Martin Sprenger, Wolfgang Rauch, Hannes Schenk, Robert Krause, Peter Willeit, Herbert Oberacher, Tracy Beth Høeg, John P A Ioannidis, Stefan Pilz

**Affiliations:** Department of Internal Medicine, Division of Endocrinology and Diabetology, Medical University of Graz, Graz, Austria; Institute for Surveillance and Infectious Disease Epidemiology, Austrian Agency for Health and Food Safety, Vienna, Austria; Institute for Surveillance and Infectious Disease Epidemiology, Austrian Agency for Health and Food Safety, Vienna, Austria; Institute of Statistics, Graz University of Technology, Graz, Austria; Institute of Social Medicine and Epidemiology, Medical University Graz, Graz, Austria; Department of Environmental Engineering, University of Innsbruck, Innsbruck, Austria; Department of Environmental Engineering, University of Innsbruck, Innsbruck, Austria; Department of Internal Medicine, Division of Infectious Diseases, Medical University of Graz, Graz, Austria; Institute of Clinical Epidemiology, Public Health, Health Economics, Medical Statistics, and Informatics, Medical University of Innsbruck, Innsbruck, Austria; Department of Public Health and Primary Care, University of Cambridge, Cambridge, United Kingdom; Ignaz Semmelweis Institute, Interuniversity Institute for Infection Research, Vienna, Austria; Institute of Legal Medicine, Medical University of Innsbruck, Innsbruck, Austria; Core Facility Metabolomics, Medical University of Innsbruck, Innsbruck, Austria; Sloan School of Management, Massachusetts Institute of Technology, Cambridge, Massachusetts, USA; Department of Clinical Research, University of Southern Denmark, Syddanmark, Denmark; Department of Emergency Medicine, University of California San Francisco, San Francisco, California, USA; Department of Medicine, Stanford University, Stanford, California, USA; Department of Epidemiology and Population Health, Stanford University, Stanford, California, USA; Department of Biomedical Data Science, Stanford University, Stanford, California, USA; Meta-Research Innovation Center at Stanford, Stanford University, Stanford, California, USA; Department of Internal Medicine, Division of Endocrinology and Diabetology, Medical University of Graz, Graz, Austria

**Keywords:** SARS-CoV-2, COVID-19, wastewater, immunization, nationwide

## Abstract

**Background:**

Postpandemic surveillance data on coronavirus disease 2019 (COVID-19) infections may help inform future public health policies regarding severe acute respiratory syndrome coronavirus 2 (SARS-CoV-2) testing, vaccinations, or other COVID-19 measures. We estimate the total SARS-CoV-2 infections in Austria after the end of the pandemic from wastewater data and utilize these estimates to calculate the average national levels of SARS-CoV-2 infection protection and COVID-19 death protection.

**Methods:**

We estimated the total SARS-CoV-2 infections in Austria after the end of the pandemic (5 May 2023, per World Health Organization) up to May 2024 from wastewater data using a previously published model. These estimates were used in an agent-based model (ABM) to estimate average national levels of SARS-CoV-2 infection protection and COVID-19 death protection, based on waning immunity estimates of infections and vaccination in previous literature.

**Results:**

We estimate approximately 3.2 million infections between 6 May 2023 and 23 May 2024, with a total of 17.8 million infections following 12 May 2020. The ABM estimates that the national average death protection was approximately 82% higher in May 2024 than before the pandemic. This represents a relative decrease of 8% since May 2023. It also shows that 95% of people in Austria were infected with SARS-CoV-2 at least once by May 2024. National infection protection remained relatively low after the onset of Omicron.

**Conclusions:**

These findings should be considered for public health decisions on SARS-CoV-2 testing practices and vaccine booster administrations.

## Teaser

Wastewater estimates of SARS-CoV-2 infections in Austria show strong COVID-19 death protection despite declining vaccinations, with only a small decrease in protection levels a year after the pandemic's official end.

Immune conferring events by severe acute respiratory syndrome coronavirus 2 (SARS-CoV-2) vaccinations and infections were both associated with significantly declining infection fatality rates (IFR) and the declaration by the World Health Organization of the end of the coronavirus disease 2019 (COVID-19) pandemic by 5 May 2023. Thereafter, active national surveillance data based on SARS-CoV-2 testing and tracking of severe and fatal COVID-19 cases are largely missing. Postpandemic surveillance data on COVID-19, however, may be required to inform future public health policies regarding SARS-CoV-2 testing, vaccinations, or other COVID-19 measures. In particular, estimates of the potential COVID-19 disease burden such as the IFR are important to guide us on how to balance the risks and benefits of any recommendation regarding COVID-19.

Estimating the number of SARS-CoV-2 infections based on wastewater data in the postpandemic phase provides a measure on the extent of immune conferring events and thus on the immunological protection against COVID-19 in the general population [1, 2]. Immunological protection conferred after SARS-CoV-2 infections, termed natural immunity, may be superior to vaccine-induced immunity as it wanes slower regarding protection against infection [3]. Importantly, immunity by SARS-CoV-2 vaccination, infection, and a combination thereof, termed hybrid immunity, all provide significant long-term protection against severe and fatal COVID-19. This protection against severe and fatal COVID-19 is long lasting with little evidence of waning, whereas protection against SARS-CoV-2 infections wanes rapidly and is thus relatively short-lived [3–6]. In line with this, SARS-CoV-2 infection rates were still relatively high towards the end of the COVID-19 pandemic, boosting immunity in the general population, whereas IFR continuously declined. How immunity to SARS-CoV-2 and IFR further evolved in the postpandemic phase is, however, largely unknown.

In this study we used a previously published model on national wastewater data to estimate the number of all SARS-CoV-2 infections that occurred in Austria after the declared end of the COVID-19 pandemic, from 6 May 2023 to 23 May 2024 [1]. In addition, we estimated the nationwide average protection against COVID-19 death and protection against SARS-CoV-2 infections by using an agent-based model (ABM), as an extension of a susceptible–infectious–recovered (SIR) model, that estimates waning immunity according to literature-based data and incorporates the estimated total infections and documented vaccinations [3–5, 7–14].

## METHODS

### Design and Analysis

We conducted a retrospective estimation of total SARS-CoV-2 infections in the entire population of Austria from 12 May 2020 to 23 May 2024, based on wastewater monitoring data [1, 2, 15]. Additionally, an ABM was constructed to estimate the temporal changes of the nationwide level of immunization in form of death protection and infection protection. Death protection and infection protection reflect how much lower the probability of death and infection, respectively, is when compared to immunity-naive individuals. This is akin to how vaccine effectiveness is often calculated (1 − hazard ratio) [16, 17]. The model was based on individual levels of death protection and infection protection after a previous infection, previous vaccination (1, 2, and 3 or more doses), a hybrid immunization (at least 1 infection and 1 vaccination) and their respective rates of waning, retrieved from the literature [3–5, 7–14]. We used the 4-month moving average death protection and regressed them onto 4-month moving average IFR estimates after April 2020 [18]. We also similarly regressed estimates from January 2022 onward, coinciding with Omicron dominance. The IFRs were estimated by dividing all COVID-19 deaths that occurred within a 4-month range around a date, by all first positive tests of an infection that occurred in that same period. To account for time lag between infection and death, we analyzed deaths as if they occurred on the first day of the last recorded SARS-CoV-2 infection that led to the death. Subsequent positive tests were counted as new infections if they occurred at least 90 days later. For the estimates we used 30-day COVID-19 mortality [19].

The study was approved by the ethics committee at the Medical University of Graz (No. 33–144 ex 20/21). Analyses were prespecified and agreed among authors before any data were analyzed. The statistical analyses and simulations were conducted using R (version 4.4.1) [20].

### Study Data

Wastewater data from the Austrian SARS-CoV-2 wastewater monitoring initiative was provided by the Austrian Federal Ministry of Social Affairs, Health, Care, and Consumer Protection for the period from 1 November 2022 to 31 May 2024. Estimates of daily active infections between May 2020 and December 2022 were provided by a previous publication [1]. Data on nationwide daily vaccinations and number of doses were publicly available for the period between 27 December 2020 and 1 January 2024 [21]. As in previous publications, documented SARS-CoV-2 infections and COVID-19 deaths were provided by the Austrian Agency for Health and Food Safety (German, Österreichische Agentur für Gesundheit und Ernährungssicherheit; AGES) and acquired through the Austrian epidemiological reporting system (German, Epidemiologisches Meldesystem; EMS) [16, 22].

### Models

#### Infection Estimation From Wastewater Data

For the estimation of total SARS-CoV-2 infections, we applied a previously published approach that estimated infections in Austria based on wastewater data from May 2020 to December 2022 [1]. The model showed high overlap with 2 different approaches estimating total infections based on IFRs and test positivity (testing rate and reported cases) [1]. Estimates of active infections between 30 April 2020 and 17 December 2022 were available from the original publication, and the model was extrapolated up to 31 May 2024, using the respective wastewater data. Usage of backpropagation for estimation of daily new infections (Supplementary Material) led to the earliest estimates of new daily infections on 12 May 2020 and latest on 23 May 2024.

A description on the wastewater monitoring data as well as the preprocessing and normalization methodology is presented in the Supplementary Material, and more details are in previous publications [1, 2, 15, 23, 24]. In short, the data was filtered, outlier corrected, averaged, and interpolated before the model estimation. The model is based on a parameter estimate that represents a combined shedding and loss factor (how many gene copies can actually be identified per infection), which the original paper estimates for multiple timeframes [1]. We adopted the last used parameter estimate (from May 2022 onward) to extrapolate the analysis up to 31 May 2024. We conducted preliminary analyses to recalibrate the parameter by varying degrees and incorporated these adjustments into the ABM that accounts for reinfection risk. Our findings indicated that reducing the parameter estimates by 25% (equivalent to increasing the estimated daily infections by 25%) produced population outcomes aligned with later seroprevalence studies (Supplementary Table 3) [25]. Consequently, we adopted these recalibrated estimates for the main analysis.

We correlated recorded infections with the estimated total infections between 17 December 2022 and 30 June 2023, as an indication of how well-documented fluctuations in infections are represented in the estimates. This marks the period in which the previous study did not estimate total infections, but infections were still officially documented. For a detailed description of the model see Supplementary Methods and the original publication [1].

#### Agent-Based Model

**Figure 1.**
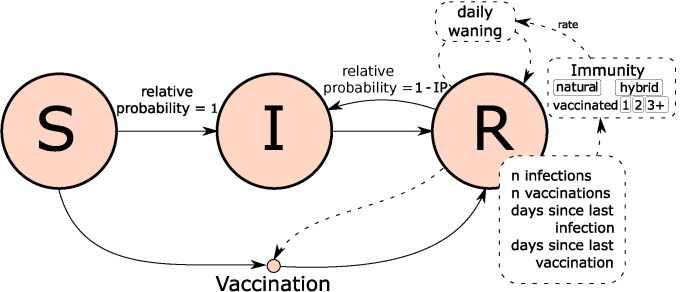
Infection protection (IP) and death protection estimation model concept. Agents can be in state S, I, or R (susceptible, infectious, recovered). Recovered agents save state information about previous events to group them into natural immunity, vaccinated with 1, 2, 3 or more doses, and hybrid immunity. Group-specific waning rates and information on days since last infection are then used to calculate the daily infection protection and death protection.

We constructed an ABM to simulate the immunity level (death protection and infection protection) of the general population of Austria between 12 May 2020 and 23 May 2024. It was conceptualized as an extended SIR model where the individual state of every agent is tracked, and previously estimated infections are probabilistically distributed based on the individual infection protection levels (individual susceptibility = 1 − infection protection) (Figure 1). We additionally included the option of vaccination to move from S to R. The state tracks number of previous infections, number of previous vaccinations, days since last infection, and days since last vaccination for every agent in R (Figure 1). From this state we categorized every agent into 1 of 5 compartments (natural immunity; vaccinated 1, 2, 3 or more times; and hybrid immunity). Agents in these categories have a nonzero infection protection, which wanes over time, the rate of which is based on published data estimates of infection protection waning in the respective category [3–5, 7–14]. Agents in S have an infection protection of zero. Infected agents are in I for 14 days before moving to their respective R compartment. During this time, they could not be categorized as either infected or vaccinated. We additionally tracked the daily individual death protection, also based on published data estimates of death protection specific waning [3–5, 10–13]. By averaging the individual infection protection and death protection, respectively, excluding currently infected individuals, we calculated the level of daily average national infection protection and death protection. Vaccinations were lagged 14 days to account for the time needed to become effective. We set the population to 9 020 000 [1].

Waning protection was implemented using a Gompertz function [4]. To estimate the parameter for the function based on waning estimates, we fitted the Gompertz curve to the waning estimates using nonlinear least squares [4].

We retrieved individual estimates of waning immunity for first, second, and third or more vaccinations, as well as estimates for previous infections and hybrid immunity from previous publications [3–5, 7–14]. For a description of waning immunity estimates including sources and values, see Supplementary Methods (Supplementary Tables 1 and 2, Supplementary Figure 1). If hybrid immunity occurred, or a subject with hybrid immunity had an additional event, protection against reinfection and death were increased to the same level irrespective of number of infections, vaccination dose, and event order [9]. Studies show much lower infection protection in the Omicron period than before. So, we researched separate estimates for pre-Omicron and Omicron periods and continuously transitioned waning estimates of individual infection protections between them throughout a month in early 2022, at the onset of Omicron [4]. There are no randomized data on the effectiveness of COVID-19 vaccines in the setting of the Omicron variant. Existing literature does not indicate that Omicron led to a decrease of protection from death; however, the methodology used to assess vaccine effectiveness against death may be inherently biased [26, 27]. For more details and a full mathematical description of the model please see the Supplementary Methods.

Our preanalyses showed that variability of estimates decreased with lower simulation scaling (Supplementary Methods, Supplementary Figure 3). Thus, we decided to run the main simulation 15 times at a scale factor of 10. Scaling was performed by dividing daily estimated infections, vaccinations, and the population by the scaling factor and rounding. The approach in this article constitutes a conceptual extension to previously published investigations on gradual waning of immunity [28–30]. We accounted for various sorts of bias like healthy vaccinee bias in the sensitivity analyses [16, 17, 31–33].

### Sensitivity Analyses

We conducted sensitivity analyses on the parameters of waning in previously infected, 1, 2, or more times vaccinated, and hybrid immunized individuals, by rerunning the simulation with waning death protection 110%, 90%, and 75% the magnitude of the original waning estimates. We also decreased infection protection estimates for the previously infected only, previously infected plus hybrid immune, and infection protection of all 3 vaccination conditions to 75% to see the effects on infection distributions. We addressed the possibility of overestimating or underestimating the number of infections after the end of the pandemic by performing the main analysis with 125%, 110%, 90%, and 75% of the estimated daily infections. To investigate the possibility of over- and underestimating infections throughout the whole pandemic, we also ran it with 125%, 110%, 90%, and 75% of the estimated daily infections. We addressed healthy vaccinee bias by running the simulation with decreased death protection for vaccinated individuals (including the 1, 2, and 3 or more vaccination categories) using 75% and 50% of the main simulation magnitude. Some literature indicates no waning of death protection in previously infected and vaccinated individuals [4, 5]. To address this possibility, we ran the simulation with alternative estimates for infection and hybrid death protection with no waning and estimates that were between these and the original estimates. Lastly, we ran the simulation without vaccinations. To decrease computational demand, we downscaled the sensitivity analyses by a factor of 200 while still running them 15 times each.

## RESULTS

### Infection Estimation From Wastewater Data

**Figure 2.**
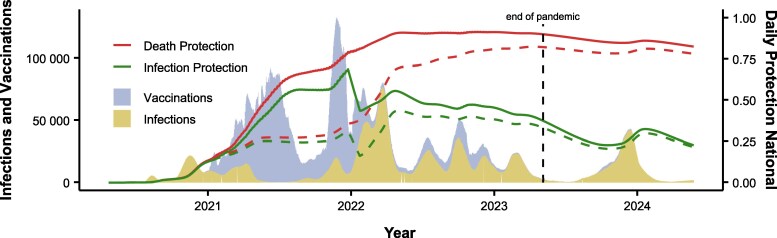
Death protection and infection protection timeline. Dashed lines are simulated estimates of protection levels without vaccinations.

The model estimated a total of approximately 3.2 million infections between 6 May 2023 and 31 May 2024. Between 12 May 2020 and 23 May 2024, 17.8 million total infections were estimated in Austria (Figure 2; see Supplementary Table 3 for exact estimates used in ABM; also see Supplementary Figure 4 and Supplementary Results for confidence intervals). Including the 20 208 999 documented vaccinations, this totals close to 38 million immune-conferring events (Figure 2). Extrapolated estimates for the period between 17 December 2022 and 30 June 2023 were correlated by *r* = 0.985 with 7-day averaged documented infections (Supplementary Figure 5).

### Agent-Based Model

**Figure 3.**
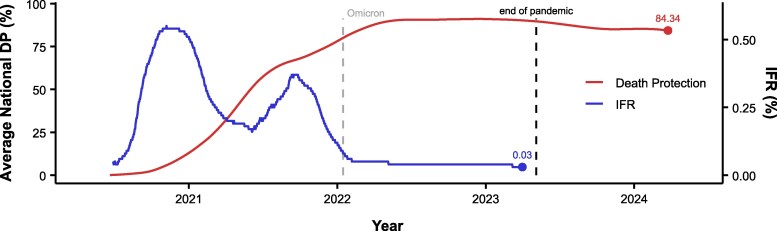
Comparison of average estimated death protection (DP) and infection fatality rate (IFR). The IFR is based on the infection estimates and 30-day mortality data. Death protection and IFR represent 4-month moving averages. Note the difference in scales between IFR and death protection.

The ABM shows the steady rise of the relative protection of the Austrian population throughout the pandemic, with ongoing high average death protection a year after the pandemic's end (Figure 2 and Figure 3). On 5 May 2023 and 23 May 2024, the estimated national average death protection was approximately 90% and 82%, respectively. Thus, in May 2024 protection was still at 92% of the level at the end of the pandemic (Supplementary Table 3).

Our model estimated that by 23 May 2024, a total of 99% of the Austrian population had experienced at least 1 immune-conferring event, including 95% with at least 1 infection (Supplementary Table 3). About 69% of the infected population had experienced multiple SARS-CoV-2 infections (Supplementary Figure 8), and 47% of the total population had their first infection before or without vaccination (Supplementary Figure 9).

IFR decreased over the course of the pandemic, especially with the onset of Omicron (Figure 3). The last estimated IFR (1 April 2023) was 0.03%. Four-month rolling window death protection predicted IFR estimates after 29 June 2020 significantly (*R*^2^ = 0.5246; *F*[1,1005] = 1104; *P* < .001). Estimates after the onset of Omicron (January 2022) were also significantly predictive of IFR (*R*^2^ = 0.7681; *F*[1,454] = 1504; *P* < .001) (Supplementary Figure 10).

### Sensitivity Analyses

Sensitivity analysis (Supplementary Table 3 and Supplementary Figures 11–16) shows that the relative change in death protection between 5 May 2023 and 23 May 2024 was never more than 10%. Investigation of possible healthy vaccinee bias (downscaling protection against death provided by 1, 2, and 3 or more vaccinations) showed little effect on national death protection by May 2024 (Supplementary Figure 14). Figure 2 (dashed lines) suggests an initial positive effect of vaccination in the population in 2021 and 2022, with little difference in death protection between vaccinated and unvaccinated by 2023 and 2024. The conditions with alternative death protection estimates (slower or no waning) showed less to virtually no waning in national death protection between May 2023 and May 2024 (Supplementary Figure 15). For all results see Supplementary Table 3 and Supplementary Results.

## DISCUSSION

We estimated that 3.2 million SARS-CoV-2 infections (about 35% of the population) occurred in Austria between 6 May 2023 and 23 May 2024. The ABM indicates high national death protection from COVID-19 throughout this period even with the low number of recent vaccinations and no data on vaccination after January 2024. Sensitivity analyses showed that these findings are mostly robust to fluctuations in parameter choice. Reducing vaccination death protection to account for a potential healthy vaccinee bias did not alter our findings that protection against death was high up to May 2024.

The findings suggest that Austria has maintained a high level of postpandemic protection against COVID-19 deaths, irrespective of vaccine boosters. The high number of estimated infections points to a high number of asymptomatic or mild cases of COVID-19, which is in line with very low IFR estimates at the end of the pandemic. This may be due to a combination of very low IFR for the currently prevailing variants plus a high level of national death protection.

Our findings should be considered for public health policy regarding COVID-19 measures such as weighing the potential benefits and potential harms of SARS-CoV-2 testing [34–37]. They also further highlight the need to critically scrutinize the continued widespread recommendation of booster vaccines. As the number of individuals who have never been infected with SARS-CoV-2 dwindles, and prior data from Austria show that booster vaccinations may have no significant effect on the COVID-19 death risk for those who were previously infected, it is critical to cautiously balance the benefits with the risks and costs of further COVID-19 vaccine doses [16]. Such considerations must not only take into account various adverse health consequences of SARS-CoV-2 infections beyond COVID-19 mortality, which may or may not be attenuated by additional vaccinations, but also the small but existing harms of vaccinations and the overall cost-effectiveness.

Results from the ABM further show that, even when accounting for possible infection overestimation, underestimation of waning, and vaccine effectiveness overestimation, average national death protection remains high. This may be in part due to the fact that protection against infection wanes faster than protection against death, which leads to new infections in individuals before their death protection can wane significantly. The very high number of asymptomatic or mild cases and the very low IFR support the hypothesis that the more time that elapses since the beginning of the COVID-19 pandemic, the more SARS-CoV-2 resembles the endemic characteristics of the other human coronaviruses [38, 39]. This may indicate public health policy regarding SARS-CoV-2 should be similar to other endemic human coronaviruses [40].

Extrapolation of total SARS-CoV-2 infection estimates were based on parameter value estimates from 2022, thus potentially limiting the validity of our estimates on the new daily infections from 2023 onward. We tried to address this in the preanalyses and sensitivity analyses, by recalibrating parameter estimates and varying estimated infections (Supplementary Figure 11). These estimates themselves are based on seroprevalence studies [41, 42]. Thus, potential errors may also be reflected in our ABM. While seroprevalence wanes and estimates late in the pandemic are potentially underestimations, the percentage of vaccinated individuals was unrepresentatively high, leading to a potential overestimation. Consequently, individual seroprevalence estimates are not perfect indicators of population-level protection; however, the referenced seroprevalence estimates and our findings show significant alignment with estimates from various other countries [43, 44].

We also do not have total infection estimates before 12 May 2020, even though this marks the period with the highest percentage of unidentified infections [41]. As these should make up a relatively small part of overall infections, we do not expect this to change our main conclusions. However, this limitation may mean the true rate of death protection is higher than reported in this article. The use of parameter estimates from late 2022 for infection estimation up to 2024 is likely imprecise, which we tried to account for by varying estimated infections in the sensitivity analyses. The model does not address possible variant-dependent variability in vaccine effectiveness, outside of the pre-Omicron and post-Omicron differentiation. General limitations in infection estimation from wastewater data and differences in preprocessing regimes have been documented in previous publications [45, 46].

The ABM has multiple limitations based on available data and assumptions. For example, there are no data available on vaccination after 1 January 2024. As such, the national protection against death may be underestimated thereafter, although vaccination rates were already exceedingly low by the end of 2023, for example, only 62 172 (0.69%) people received a vaccine dose in November or December 2023 (Supplementary Figure 2). We did not consider variations in effectiveness of different vaccine types.

Vaccine effectiveness estimates were, out of necessity, based on observational data, which are prone to multiple types of biases. Thus, there is inherent uncertainty about the magnitude and duration of infection protection and death protection during the entire study period. We are not able to determine with our current methodology the exact extent to which vaccinations, prior infections, and intrinsic virulence of the Omicron variant have independently affected the currently very low IFR (0.03%).

Furthermore, the model does not account for probabilities of infection and rates of waning moderated by age, comorbidities, or other risk factors. As such, average national protection against death may be high, but some individuals may still have low levels of protection. It is unclear though whether vaccine boosters by late 2024 and thereafter might be effective even in this population subsegment. The model also does not account for changes in population based on births, deaths, immigration, emigration, or aging.

## CONCLUSION

This national investigation in Austria based on wastewater data estimates a high number of SARS-CoV-2 infections and thus a high level of immunological protection against COVID-19 deaths after the official end of the COVID-19 pandemic (ie, 5 May 2023). In light of previous studies on the potential harm of SARS-CoV-2 testing and studies showing booster vaccinations may not significantly increase protection from COVID-19 death in previously infected individuals, our findings support the ongoing recommendation against widespread SARS-CoV-2 testing and boosters for large parts of the general population [16, 34–37]. Further research on booster effectiveness, average national protection levels, and harm of testing, as well as economical pressure of ongoing COVID-19 policies on the health care system, is needed to address the net impact of these policies.

## Supplementary Material

jiaf054_Supplementary_Data

## Supplementary Data

Supplementary materials are available at *The Journal of Infectious Diseases* online (http://jid.oxfordjournals.org/). Supplementary materials consist of data provided by the author that are published to benefit the reader. The posted materials are not copyedited. The contents of all supplementary data are the sole responsibility of the authors. Questions or messages regarding errors should be addressed to the author.

## Notes

***Acknowledgments*.** The authors thank all persons and organizations involved in data collection.

***Author contributions.*** U. R. and S. P. conceptualized the study with contributions from J. P. A. I., and wrote the original draft of the manuscript. S. P. acquired funding and administered the project. U. R. and H. S. wrote the software. A. C., L. R., W. R., and H. O. curated data. U. R. performed the formal analyses and visualization. U. R., H. O., and L. R. performed the validation. All authors contributed to writing, reviewing, and editing the manuscript, and approved the final version before submission.

***Financial support.*** This work was supported by the Austrian Science Fund (grant number KLI 1188). For this study, data was used from the National SARS-CoV-2 Wastewater Monitoring Program, which is financed by the Austrian Federal Ministry of Social Affairs, Health, Care and Consumer Protection who has the sole right of use for the data.

***Data sharing.*** The data that support the findings of this study are available upon request with approval needed from the Austrian Federal Ministry of Social Affairs, Health, Care, and Consumer Protection and Austrian Agency for Health and Food Safety (German: Österreichische Agentur für Gesundheit und Ernährungssicherheit; AGES).
