# Supplementary material for: Estimates of SARS-CoV-2 Infections and Population Immunity After the COVID-19 Pandemic in Austria: Analysis of National Wastewater Data
Source: J Infect Dis. 2025 Feb 18;231(5):e921–8. doi: 10.1093/infdis/jiaf054 (PMC12128050; doi:10.1093/infdis/jiaf054)
Supplement: jiaf054_Supplementary_Data [file jiaf054_supplementary_data.docx]

**Supplements: Post pandemic estimates of SARS-CoV-2 infections and population immunity in Austria: Analysis of national wastewater**

**Table of Contents**

[**Supplementary Methods** 2](#_Toc180140413)

[**Wastewater** **Model** 2](#_Toc180140414)

[**Data** **Description** 2](#_Toc180140415)

[**Data** **Preprocessing** 2](#_Toc180140416)

[**Model** **Description** 2](#_Toc180140417)

[**Backcasting** 3](#_Toc180140418)

[**ABM Model** 4](#_Toc180140419)

[**Model Description** 4](#_Toc180140420)

[**Gompertz Curve Parameters** 7](#_Toc180140421)

[**Vaccination** 12](#_Toc180140422)

[**Effects of Scaling on Variance between Runs** 12](#_Toc180140423)

[**Supplementary** **Results** 13](#_Toc180140424)

[**Infection Estimation** 13](#_Toc180140425)

[**Agent-based model** 14](#_Toc180140426)

[**IFR** **Regression** 15](#_Toc180140427)

[**Sensitivity Analyses** 16](#_Toc180140428)

[**Sensitivity Figures** 17](#_Toc180140429)

[**Supplementary Discussion** 23](#_Toc180140430)

[**Limitations** 23](#_Toc180140431)

[**References** 26](#_Toc180140432)

# **Supplementary Methods**

## **Wastewater** **Model**

### **Data** **Description**

The data from April 30, 2020 to December 17, 2022 were taken from Rauch et al., (2024).^1^ The extrapolation of this approach to May 31, 2024, is also based on this paper.

The dataset on wastewater data provided by the Austrian Federal Ministry of Social Affairs, Health, Care and Consumer Protection spanned the period from October 30, 2022, to May 31, 2024. It included 48 wastewater treatment plants (WWTP) from 2023 onward (24 before), with 7039 measurements total. These treatment plants cover approximately 58% of the Austrian population. Wastewater samples were collected twice a week.

Viral concentration is determined and processed as previously explained.^2^ Additionally, different hydrochemical parameters were used for characterizing the catchment population of the monitored wastewater treatment plants. For the chemical oxygen demand (COD) estimates, per capita equivalents were calculated using 120 g/d/person. Ammonia-nitrogen (NH4-N) estimates were calculated using 8.0 g/d/person. Total-nitrogen (Ntot) estimates were calculated using 11.0 g/d/person.^3^

### **Data** **Preprocessing**

For a detailed description of preprocessing see previous publications.^1,2^
To compensate for inherent measurement noise, Rauch et al. (2021) suggest the approach to exclude outliers if the flow volume (Q) exceeds the 90 percentile of the long term recorded inflow data of a WWTP (needs at least a year of data points).^4^

Estimates are normalized based on population-size markers, to compensate for population fluctuations within a catchment area. Following Arabzadeh et al. (2021), we used NH4-N prioritised over COD and Ntot.^3^ We computed the daily weighted averages of viral load levels per federal state. The weights correspond to the design capacity of the respective WWTPs, prioritising large plants over smaller ones. The design capacity of each WWTP is a parameter, that serves as a weighting factor when computing the weighted average of multiple measurements in spatial aggregation. In principle, the preferred weighting factor is the exact catchment population. However, this information is currently unavailable to us.

This results in a scattered time-series from WBE measurements that are not gapless on a daily basis. To distribute the timeseries data equally both, up- and downsampling approaches are viable.^5^ Here we used upsampling to get daily estimates by linearly interpolating gaps before applying data smoothing. Lastly, data filtering techniques are applied to reduce the signal noise and provide a mechanism to obtain the underlying information of the signal.

### **Model** **Description**

The measured virus load at the monitoring point is related to the population drained with the sewer system:

$$L_{virus}=\frac{c_{virus}*Q}{N}$$

Where L_virus_ is the population normalized virus load in gene copies/**p**erson/**d**ay, Q is the flow volume in L/d, c_virus_ is the virus concentration in the sample in copies/L and N is the catchment population.

Under the assumption that each infected person is shedding a certain load of gene copies per day (L_shed_ in gene copies/P/d) into the sewer system and additionally introducing a general loss term f_loss_ we get:

$$I\left( t+t_{lead} \right)=\frac{L_{virus}(t)\times N}{L_{shed}\times f_{loss}}=\frac{L_{virus}(t)\times N}{L_{corr}}$$

Where *I* is the number of infected individuals in the watershed, t_lead_ is the time lead and f_loss_ is a dimensionless loss factor.

Rauch chose t_lead_ = 7 in the original publication, based on cross correlation with documented infections.^1^ We found that the cross correlation of our estimates was highest with t_lead_ = 0, so we used that. Rauch and colleagues estimated different L_corr_ values for different timeframes. We chose to apply their estimate for the most recent timeframe (L_corr_ = 10^10.090). The population was set to *N* = 9.02 × 10^6^. As these values represent currently infected, we needed to apply a backcasting algorithm to estimate daily new infections.

### **Backcasting**

The backcasting methodology used here is designed to estimate daily infections based on daily active cases. The key assumption is that an infection lasts 14 days on average.

We first smoothed the estimated undocumented daily infection counts via 14 days centred moving average. The core of the backcasting process involves iteratively refining the infection level estimates, which were initially based on the smoothed data. Potential estimation errors from variability in counting method was addressed by setting negative testing values to 0. Lastly, after refining the estimates, a secondary smoothing step was applied to the calculated infection values.

## **ABM Model**

### **Model Description**

Our novel ABM is an extension of the classic SIR framework that accounts for multiple immunity states, vaccination, and time-dependent waning immunity. It tracks recovery states for infections, different vaccination statuses and hybrid immunity, allowing for a nuanced representation of population-level immunity dynamics. The model uses a Gompertz function to simulate waning immunity based on time since last infection or vaccination, providing a novel approach to long-term epidemic modelling of national immunity levels.

*Note*: Conceptually this model is closer to the literature on extended SIRS model (Susceptible-Infected-Recovered-Susceptible) than on SIR frameworks.^6,7^ We decided to not introduce the possibility to move from R so S for multiple reasons: (1) mathematically the Gompertz function asymptotically approaches its upper limit. Thus, waning immunities would never truly be back to base level after an event. This may be addressed by setting a range at which immunity can be considered to have reached 0. (2) In the context of our data, it seems unreasonable that more than a few agents avoided infections for long enough for the waning to progress this far. (3) Most importantly, immunity levels against infection may wane enough to be comparable to agents in S, but waning of protection from death is estimated to be so slow that it won’t reach 0 in the timeframe of this study (more than 4 years). Thus, categorizing previous R grouped individuals into S, would be a misrepresentation of the underlying state. In other words: We do not think that grouping individuals with no infection protection (IP) but some level of death protection (DP) into the category “Susceptible” is a sensible practice. This should be (re-)considered by researchers that plan to use this model on longer timescales, with lower infection rates, on different diseases or with different waning functions.

**Model Structure:**

For each agent *i* at time *t*:

1. C_i_(t): State variable (0: Susceptible, 1: Infected, 2: Recovered)
2. I_i_(t): Infection status (0, 1+)
3. V_i_(t): Vaccination status (0, 1, 2, or 3+ doses)
4. T_i_(t): Time since last immunity-affecting event
5. P_R,i_(t): Protection level against infection
6. P_D,i_(t): Protection level against death
7. IM_i_(t): Immunity type
   1. no immunity (0)
   2. infection (1)
   3. vaccination doses
      1. one (2)
      2. two (3)
      3. Three or more (4)
   4. hybrid immunity (5)

**Model Implementation**

1. Initialize all N agents with all states = 0.
2. For each time step t:
   1. Update immunity levels P_R,i_(t) and P_D,i(_t) for all agents based on their immunity type IM_i_(t).
   2. Apply the **Infection Process** using infection data.
   3. Apply the **Vaccination Process** using vaccination data.
   4. Apply the **Recovery Process** for infected agents.
   5. Update immunity timer T_i_(t) for all agents.
   6. Collect population-level statistics, including distributions of immunity types and protection levels.

**Immunity Dynamics**

Gompertz-based waning immunity: Immunity wanes according to a Gompertz function, depending on immunity level i:

$$G_{i(T_{i(t)})}=a_{i}*e^{{-b}_{i}*e^{{-c}_{i}*T_{i}(t)}}$$

Protection from infection:

$$P_{R,i}(t)=a_{R,{IM}_{i}}*e^{{-b}_{R,{IM}_{i}}*e^{{-c}_{R,{IM}_{i}}*T_{i}(t)}}$$

Protection from death:

$$P_{D,i}(t)=a_{D,{IM}_{i}}*e^{{-b}_{D,{IM}_{i}}*e^{{-c}_{D,{IM}_{i}}*T_{i}(t)}}$$

**Immunity Type Update Rules**

Upon infection:

$${If V}_{i}\left( t \right)=0: {IM}_{i}\left( t+1 \right)=1$$

$${If V}_{i}\left( t \right)>0: {IM}_{i}\left( t+1 \right)=5$$

Upon vaccination:

$${If R}_{i}\left( t \right)=0 \vee{If V}_{i}\left( t \right)=0: {IM}_{i}\left( t+1 \right)=2$$

$${If R}_{i}\left( t \right)=0 \vee{If V}_{i}\left( t \right)=1: {IM}_{i}\left( t+1 \right)=3$$

$${If R}_{i}\left( t \right)=0 \vee{If V}_{i}\left( t \right)\geq2: {IM}_{i}\left( t+1 \right)=4$$

$${If R}_{i}\left( t \right)>1: {IM}_{i}\left( t+1 \right)=5$$

**Infection Process**

For each time step *t*:

1. Get the number of new infections I_new_(t) from estimate data.
2. Create a pool of potentially infectable individuals:

$$Pool=\left\{ i | C_{i}\left( t \right)=0 or C_{i}(t)=2 \right\}$$

3. For each new infection (from 1 to I_new_(t)):
4. Calculate selection probabilities for each individual i in Pool:

$${prob}_{i}=\frac{(1-P_{R,i}\left( t \right))}{\sum_{j} (1-P_{R,j}\left( t \right))} for all j \in\mathrm{Pool}$$

5. Randomly select an individual based on these probabilities
6. Update selected individual:

$$C_{i}\left( t+1 \right)=1$$

$$I_{i}\left( t+1 \right)=I_{i}\left( t \right)+1$$

7. Update the immunity type IM_i_ (t+1) according to Immunity Type Update Rules
8. Reset immunity timer T_i_(t+1) = 0

**Vaccination Process**

For each time step t:

1. Get the number of new vaccinations for each dose $V_{\mathrm{new}_{x}}(t)$, where x=1,2,3,4+ from vaccination data.
2. For each vaccination dose x from 1 to 4+:
   1. Create a pool of eligible individuals for each dose:
      ${If x=1: Pool}_{V_{x}}=\left\{ i | V_{i}\left( t \right)=0 \mathrm{and} C_{i}(t)\neq I \right\}$
      ${If x=2: Pool}_{V_{x}}=\left\{ i | V_{i}\left( t \right)=1 \mathrm{and} C_{i}\left( t \right)\neq I \right\}$
      ${If x=3: Pool}_{V_{x}}=\left\{ i | V_{i}\left( t \right)=2 \mathrm{and} C_{i}\left( t \right)\neq I \right\}$
      ${If x=4+: Pool}_{V_{x}}=\left\{ i | V_{i}\left( t \right)\geq3 \mathrm{and} C_{i}\left( t \right)\neq I \right\}$
   2. For each new vaccination of dose x (from 1 to $V_{\mathrm{new}_{x}}(t)$):i. If $\mathrm{Pool}_{V_{x}}$ is not empty:
      1. Randomly select an individual i from $\mathrm{Pool}_{V_{x}}$
      2. Update vaccination status: V_i_(t+1) = V_i_(t) + 1
      3. Update immunity type IM_i_(t+1) according to Immunity Type Update Rules
      4. Reset immunity timer: T_i_(t+1) = 0
      5. Update protection levels P_R,i_(t+1) and P_D,i_(t+1)
3. Update population-level vaccination statistics

**Recovery Process**

For each agent i with I_i_(t) = 1:

If the agent has been infected for 14 days:

1. Set C_i_(t+1) = 2
2. Update immunity type IM_i_(t+1) according to Immunity Type Update Rules
3. Reset immunity timer T_i_(t+1) = 0

### **Gompertz Curve Parameters**

The parameter choice for the national protection from infection and death model was based on multiple previously published estimates.^8–18^ Estimation of Gomperz Curves Parameters were performed for pre-Omicron Era and Omicron Era. Protection was continuously changed from the pre-Omicron curve estimates to the Omicron curve estimates between December 25^th^ 2021 and January 25^st^ 2022. Different estimates for hybrid immunity showed consistently higher values than from a previous infection only.^12^ Interestingly, all of them showed relatively faster waning, this inevitably leads to a lower immunity level for hybrid immune than from previous infections. As waning estimates do are usually not performed or reliable on the time scale of multiple years, we expect that the hybrid immunities are approaching the immunity of previously infected. As such we set the lower bound for hybrid immunity at the respective waning immunity of the previous infected immunity (Figure S1).

#### **Pre-Omicron Era**

Table S1 show the waning estimates with their respective time points (in days) and the respective estimated start values and the reference from which this value was taken. Note that waning values of protection against infection were continuously reduced between December 25^th^ 2021 and January 25^st^ 2022 to Omicron period levels. This transition is not shown in the following figures as transition is infection time specific (Figure S1). The most reliable estimates on vaccination data in pre-Omicron Era were available for vaccine dose 2 (primary vaccination). As such we used point estimates for vaccination dose 1 and 3 to scale the dose 2 waning curve appropriately.


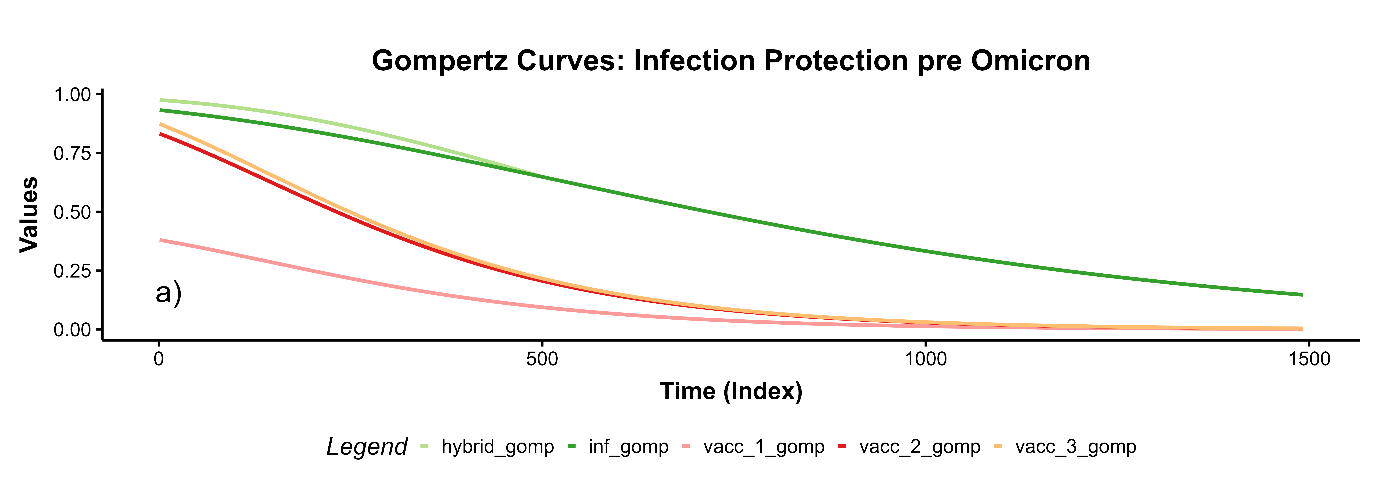
**
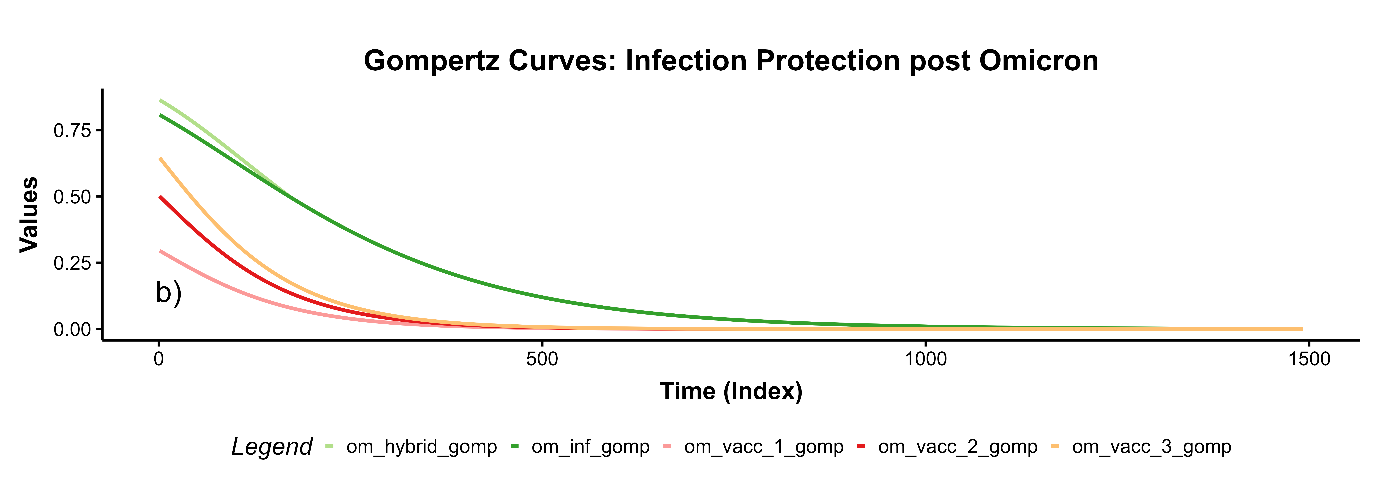

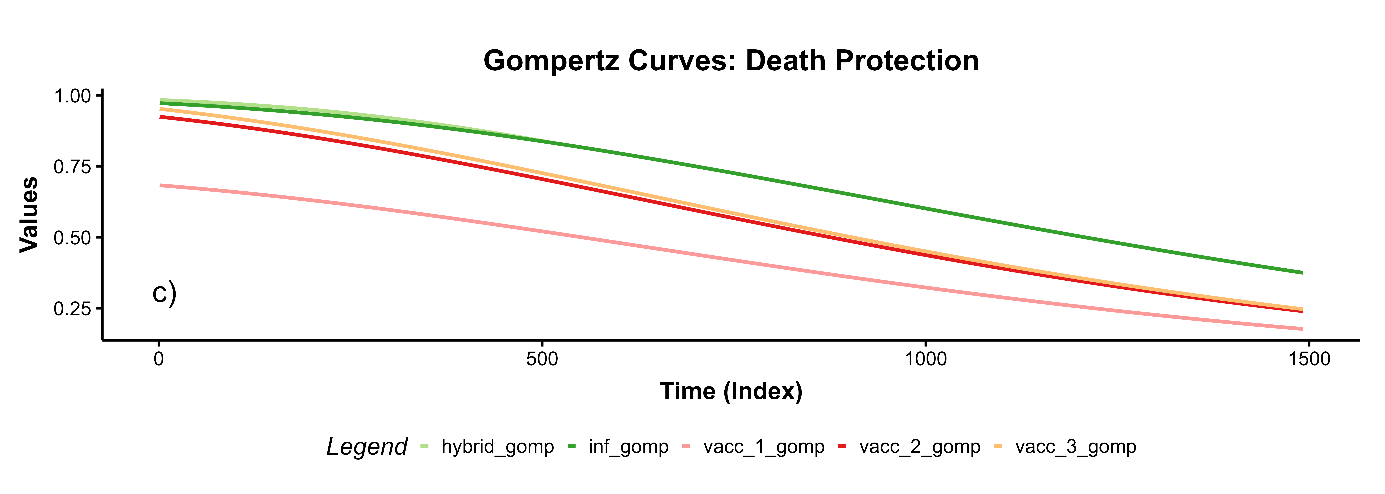

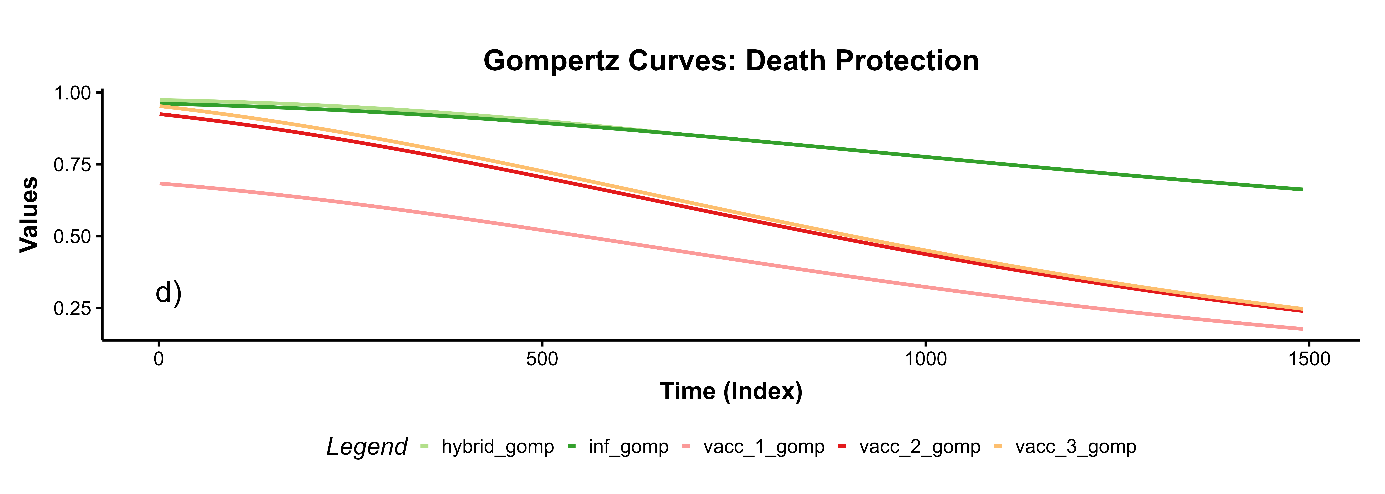
**
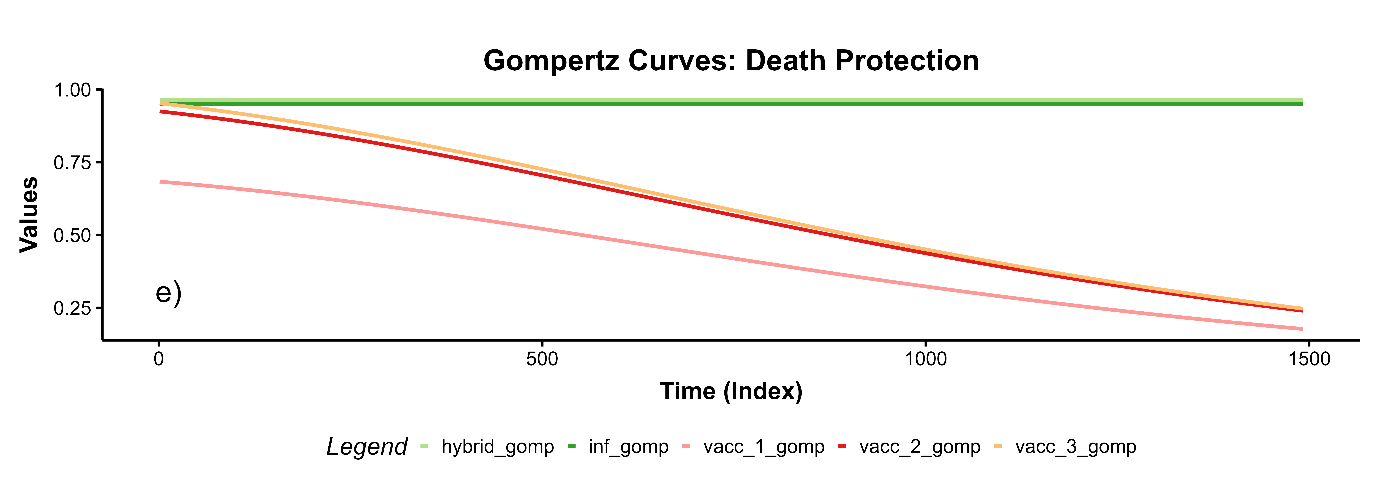
**Figure S1:** Waning curves for protection against infection pre-Omicron (a), protection against infection in Omicron (b), main protection against death (c), in-between alternative and main protection against death estimates (infection and hybrid immunity) (d) and alternative protection against death estimates (e). Hybrid immunity wanes faster than protection from a previous infection. We assumed that hybrid immunity wanes to the level of infection from a previous protection and then stays at that level.

#### **Post-Omicron Era**

The most reliable estimates on vaccination data in Omicron Era were available for vaccine dose 3 (booster vaccination). As such we used point estimates for vaccination dose 1 and 2 to scale the dose 3 waning curve appropriately (Figure S1b).

While some studies show small decreases in protection against death of previously infected in Omicron time periods, compared to pre-Omicron periods these estimates have two problems.^19^ First, the number of deaths in Omicron periods is so low, even for people without immunity, that a single death can already cause smaller protection estimations than in previous variants. Second, and more importantly, at this point of the pandemic, partially driven by the Omicron itself, the number of hidden infections is so high that even in test-negative designs the non-infected population is likely permeated by previously infected persons. Thus, indirectly decreasing the protection estimate. As most of the DP values for previously infected, are still extremely high, we thus concluded that the difference between pre-Omicron and Omicron DP estimates is likely to be an artefact. As such we did not adjust DP for previously infected, vaccinated or hybrid immune individuals at the onset of Omicron.

| **Table S1: Estimates of infection protection waning rates, references and reasoning of Gompertz functions.** | | | | |
| --- | --- | --- | --- | --- |
|  | **Estimated protection at day 1 (in percent)** | **Values (days: protection(%))** | **Reference** | **Comment** |
| **pre-Omicron IP** |  |  |  |  |
| waning after infection | 93.24 | 14: 90.5, 487: 70, 669: 50 | Chemaitelly et al., 2022^8^ | These are projected estimates |
| waning after 1 vaccination dose | 38.01 |  | Chemaitelly et al., 2021^9^ | We scaled the Gompertz curve estimates of 2 vaccination dose waning by the ratio between initial protection of 1 dose and 2 doses as given by Chemaitelly et al., 2021; 36.8% / 80.5%=0.457 |
| waning after 2 vaccination doses | 83.18 | 30: 80.5, 183: 54.6, 274: 45.9 | Menegale et al., 2023^10^ |  |
| waning after 3 or more vaccination doses | 87.0 |  | Braeye et al., 2023^11^ | We scaled the Gompertz curve estimates of 2 vaccination dose waning by the ratio between initial protection of 3 doses and 2 doses as given by Braeye et al., 2023; 87% / 83.18% = 1.05 |
| waning after hybrid immunity | 97.53 | 30: 96.58, 91: 96.1, 152: 90.25, 213: 89.17 | Goldberg et al., 2022^12^ | Goldberg provides rate ratios with 2 vaccine doses as a reference value. We used the relative protection of 2 vaccine doses stated above to calculate these values from the rate ratios of hyrbid immunity. |
| **Omicron IP** |  |  |  |  |
| waning after infection | 80.77 | 40: 77.3 , 126: 53.0, 196: , 45.5 266: 37 , 336: 37 , 406: 37 | Covid-19 Forecasting Team, 2023^13^ | Used values from Table S2 category: Protection against Omicron  BA.2 reinfection |
| waning after 1 vaccination dose | 22.88 |  | Chemaitelly et al., 2021^9^ | We scaled the Gompertz curve estimates of 2 vaccination dose waning by the ratio between initial protection of 1 dose and 2 doses as given by Chemaitelly 2021 (pre Omicron ratio); 36.8% / 80.5%=0.457 .We used the same ratio as pre Omicron as to our knowledge, there are no studies on effectiveness of partial vaccination against infection in the Omicron period. |
| waning after 2 vaccination doses | 50.06 | 30:44.4, 182: 20.7, 274: 13.4 | Menegale et al., 2023^10^ | These values present the estimates to calculate the value at day 1, not the values used for the actual Gompertz curves. We used the here estimated value to calculate a scaling from 3 vaccine doses. Menegale provides waning estimates for two doses, but these are likely biased to people that did not get a third vaccination dose. This is also indicated by the waning of IP which is much slower in the estimate of 2 doses than the estimate of 3 doses; 50.06% / 64.6% = .775 |
| waning after 3 or more vaccination doses | 64.6 | 30: 55.4, 183: 36.0, 274: 28.9 | Menegale et al., 2023^10^ |  |
| waning after hybrid immunity | 86.38 | 30: 80.1, 61: 74.8 91: 68.6 , 122: 61.6 183: 46.5 | Bobrovitz et al., 2023^14^ | We used the values for hybrid immunity (first booster vaccination) from Table 2. As these match more coherently with the waning after infection estimations, and are likely more reflective of omicron effects. |
| Note: the inverse of the protection value (susceptibility) is used to estimate the Gompertz function | | | | |
| Figure S1 shows the respective Gompertz curves  IP = Infection Protection | | | | |

| **Table S2: Estimates of death protection waning rates, references and reasoning of Gompertz functions** | | | | |
| --- | --- | --- | --- | --- |
|  | **Estimated protection at day 1 (in percent)** | **Values (days: protection(%))** | **Reference** | **Comment** |
| **protection against death** |  |  |  |  |
| waning after infection | 97.27 | 40: 97.7, 126: 95.9, 196: 92.9, 266: 91.0, 336: 88,7, 406: 88,7 | Covid-19 Forecasting Team, 2023^13^ | Used values from Table S2 category: Protection against ancestral, Alpha, and Delta severe disease |
| waning after 1 vaccination dose | 68.35 | x | Rahmani 2022^15^ | To our knowledge, there are no papers on waning of a single vaccination dose. Thus, we scaled the Gompertz curve estimates of 2 vaccination dose waning by the ratio between "initial protection" of 1 dose and 2 doses as found by Rahmani 2022 (68%/92%=0.739%) |
| waning after 2 vaccination doses | 92.48 | 28: 91.0, 131: 91.0, 154: 85.0, 182: 86.0 | Wu 2023^16^ |  |
| waning after 3 or more vaccination doses | 94.89 | 18: 88.0, 45: 86.0, 75: 80.0, 105: 82.0 | Grewal 2023^17^ | Studies on waning of third vaccine dose (first booster dose) show similar values to the second vaccination dose, but consistently faster waning. We expect that this is due to the period and nature of the conducted studies. As Omicrons increased immune evasion led to an extremely high number of cases, it is likely that the number of unidentified cases was also high. Leading to waning estimates that are based on group comparisons where more and more control group members actually had a previous infection. Thus, we decided to use the estimated initial death protection(day1) from Berec 2022^18^: 94.89 (all estimates 31: .92; 91: .93; 182: .90, 243: .83) and scale waning estimates given by two vaccine doses to its level (94.89 / 92.48 = 1.03). |
| waning after hybrid immunity | 98.39 | 30: 98.0, 61: 97.6, 91: 97.2, 122: 96.7, 183: 95.3 | Bobrovitz 2023^14^ | We used the values for hybrid immunity (first booster vaccination) from Table 2. The other option (hybrid immunity (primary series vaccination)) actually shows increasing protection over 12 months. Probably due to noise. |
| **alternative estimates** |  |  |  |  |
| waning after infection | 95.00 | 122: 93.4, 213: 94.3 274: 94.2, 304: 98.1 | Chemaitelly 2022^8^ | Estimates as seen in Figure 2c. We only used estimates that had at least one death. We did not use the 14+ month estimate as we do not have a medium estimate on the days this category encompasses |
| waning after hybrid immunity | 96.39 | 30: 95.7, 61: 95.9, 91: 96.0, 122: 96.2, 183: 96.5, 274: 97, 365: 97.4 | Bobrovitz 2023^14^ | Used the "primary series vaccination" estimates |
| Note: the inverse of the protection value (susceptibility) is used to estimate the Gompertz function | | | | |
| Figure S1 shows the respective Gompertz curves | | | | |

### **Vaccination**

**
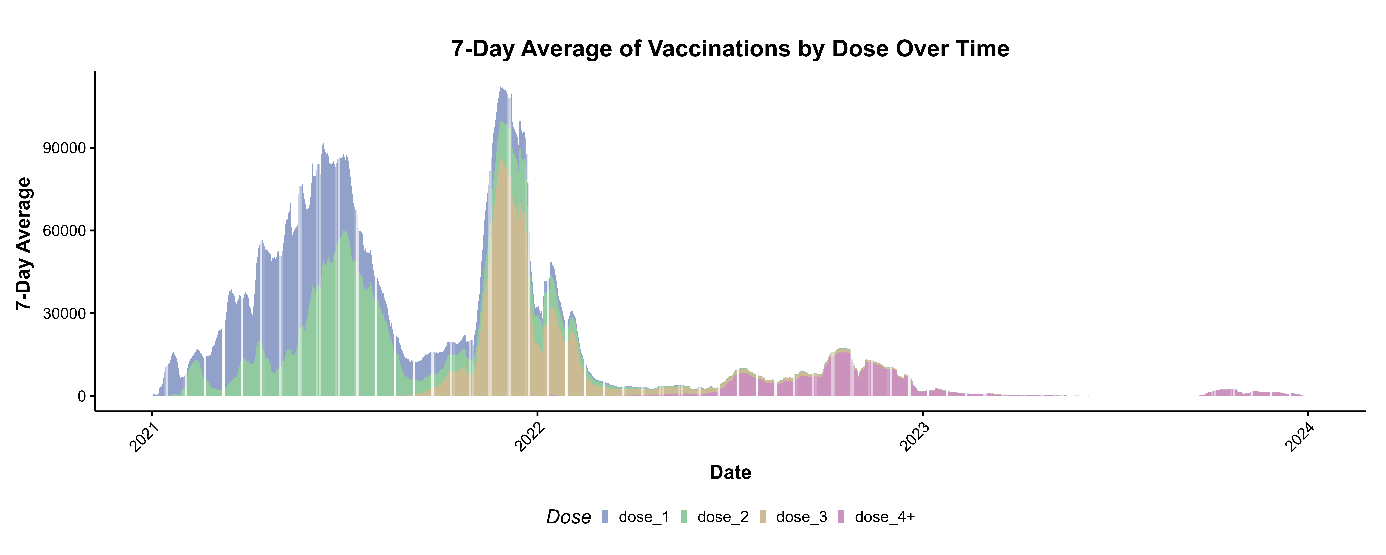
Figure S2:** (7 Day Average) Vaccinations by Dose

### **Effects of Scaling on Variance between Runs**

Early scaled analyses showed clearly that variations in IP and DP between multiple runs are small and keep decreasing with lower scaling. We calculated Root Mean Square Error (RMSE) to quantify the difference between multiple runs and based on these ever-decreasing variations (Figure S3),^20^ we concluded that using a low number of runs with a relatively smaller scaling is preferable (for computational viability) to higher scale, high run number averages.


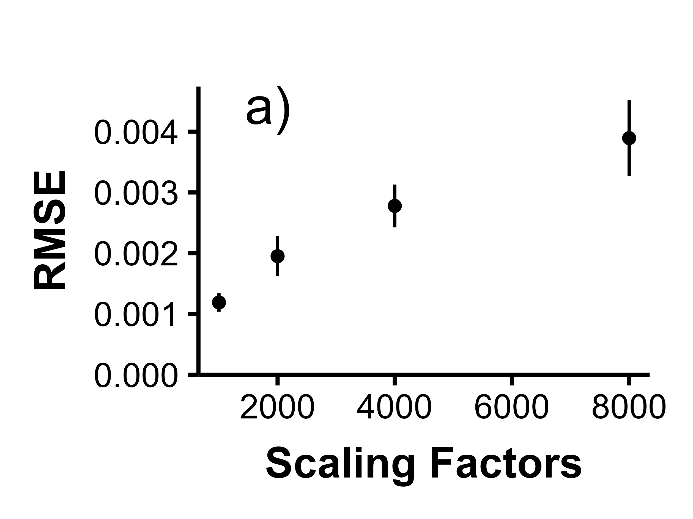

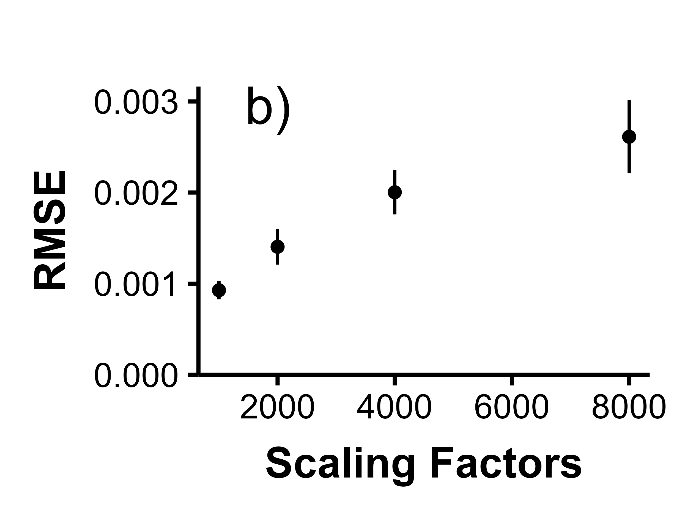


**Figure S3:** RMSE of DP (a) and IP (b) from 15 runs for different scaling factors.

# **Supplementary** **Results**

## **Infection Estimation**

As the original publication provided lower and upper bound estimates (5% and 95%) for the Lcorr parameter. As with the main analysis data, the estimates for these bounds prior to December 17, 2022 were acquired directly form Rauch et al.,(2024).^1^ After rescaling as explained in the main manuscript, we estimated a total of 18,701,194 and 17,165,579 infections for the 5% and 95% bounds respectively (Figure S4).


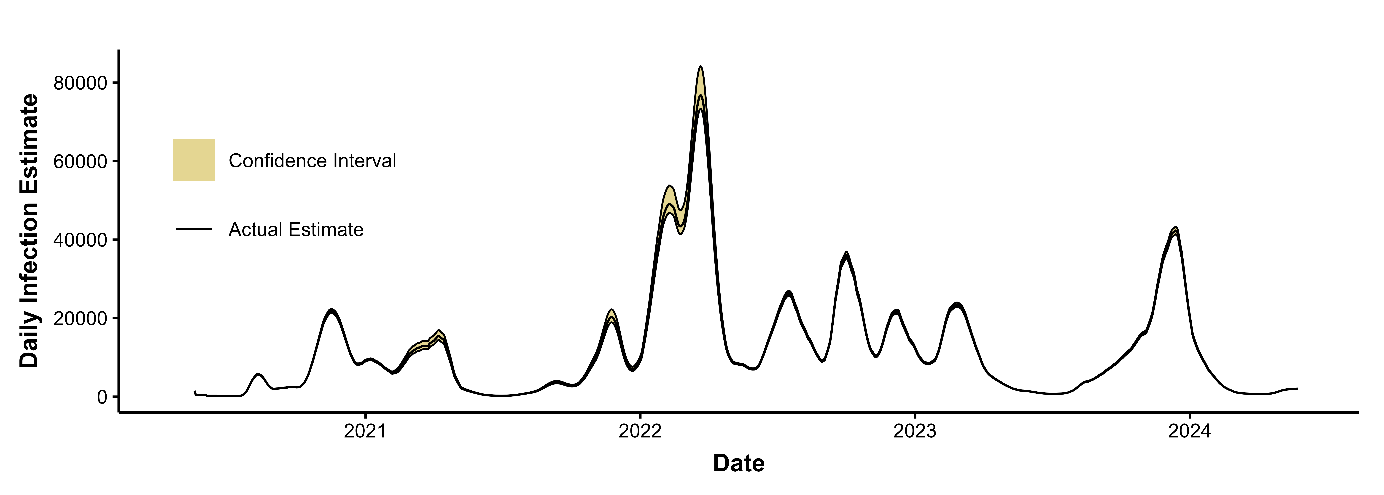


**Figure S4**: Daily infections estimated from wastewater data including lower and upper bounds as Confidence Intervals.

The wastewater model follows the trend of documented infections (7 day average) even in timeframes it was not previously applied to (Figure S5). The correlation between these two is r = .985 indicating perfectly matching trends.

**Figure S5:** Documented and estimated infections between December 17, 2022 and June 30, 2023. Documented infections were averaged over 7 days.


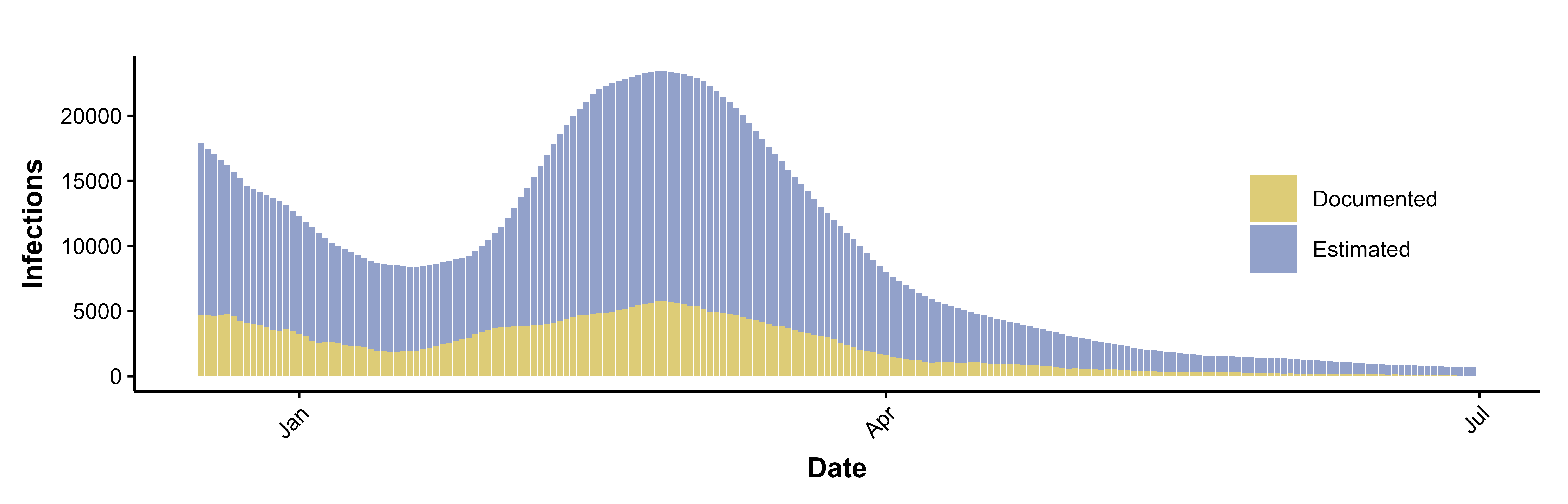


## **Agent-based model**


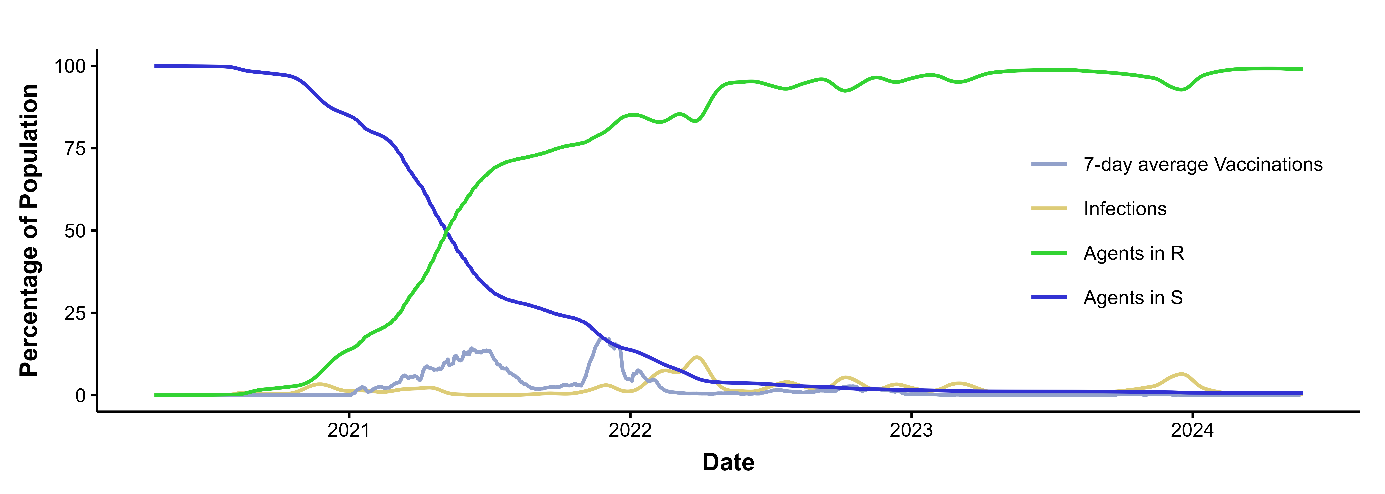
**Figure S6:** Time series of S, R, daily infections and daily vaccinations.

**Figure S7:** Percentage of people with no infection, stratified by number of vaccinations, by day.


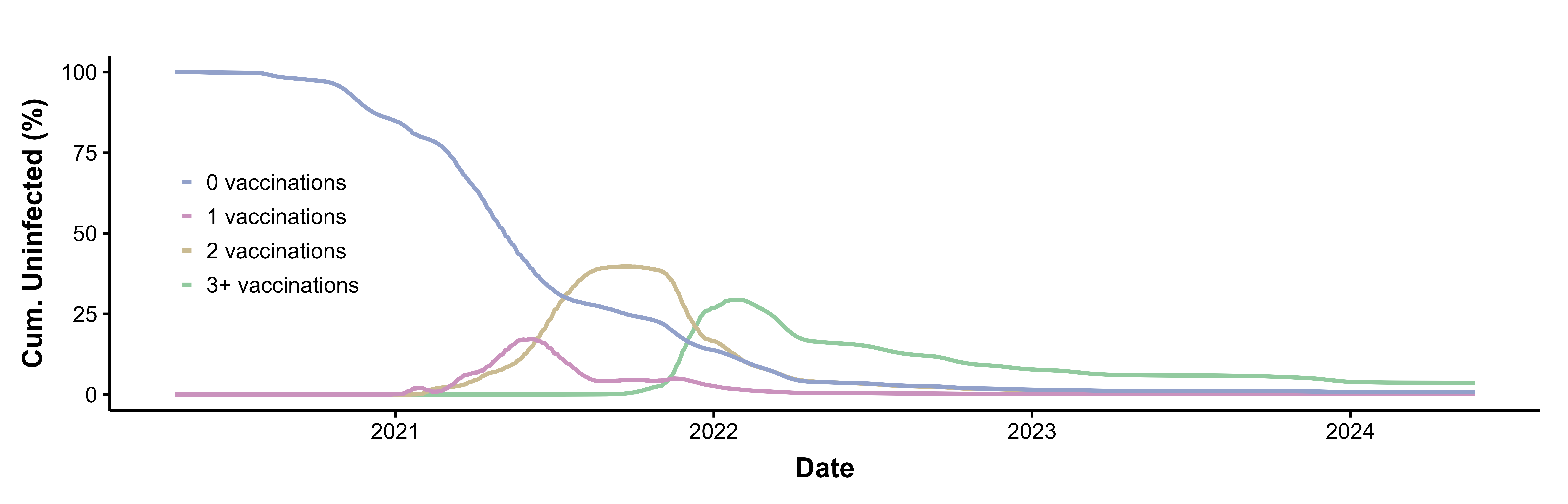


**Figure S8:** Percentage of people with at least one infection and stratified by number of infections, by day.


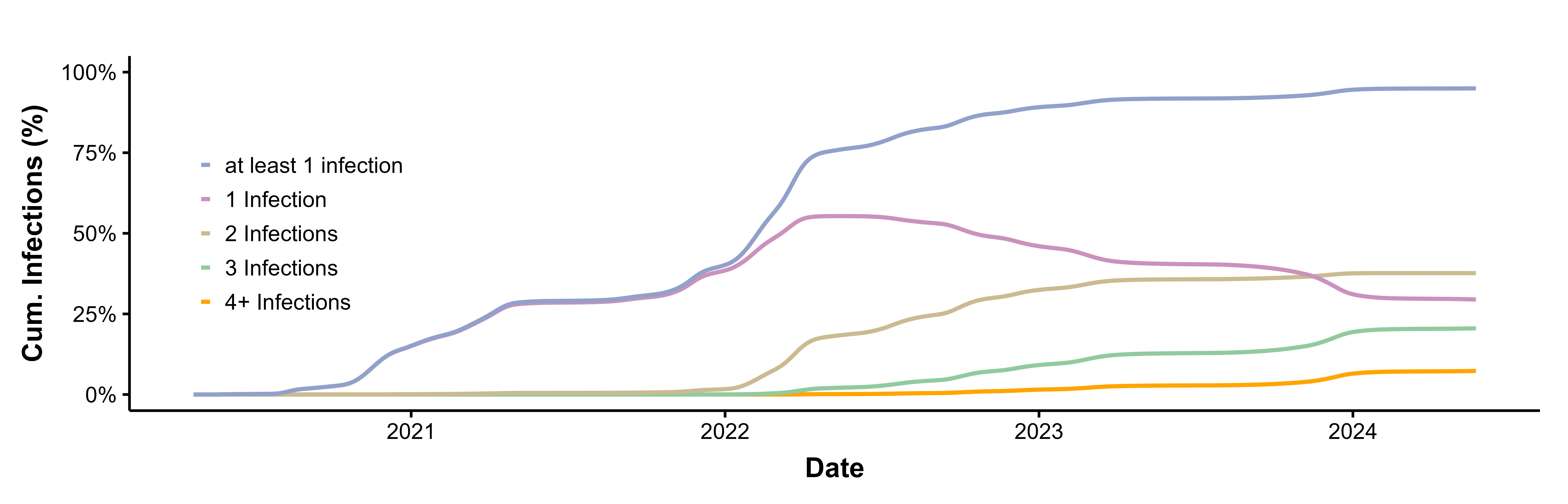


**Figure S9:** Cumulative percentage of population, stratified by vaccination status on day of first infection.


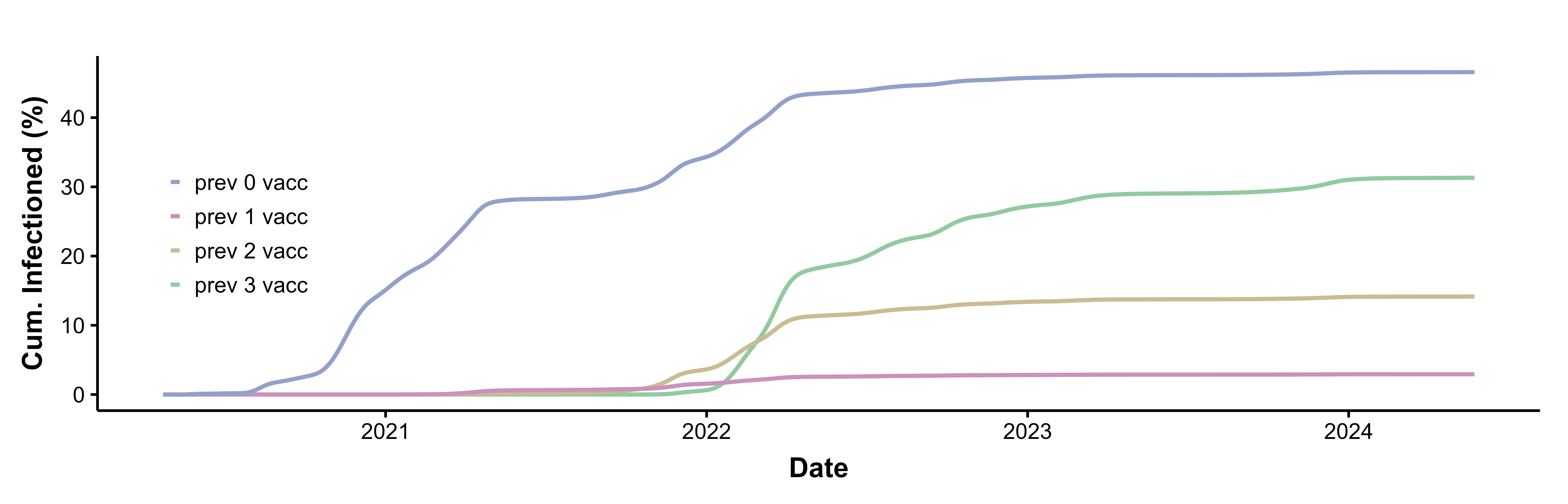


## **IFR** **Regression**


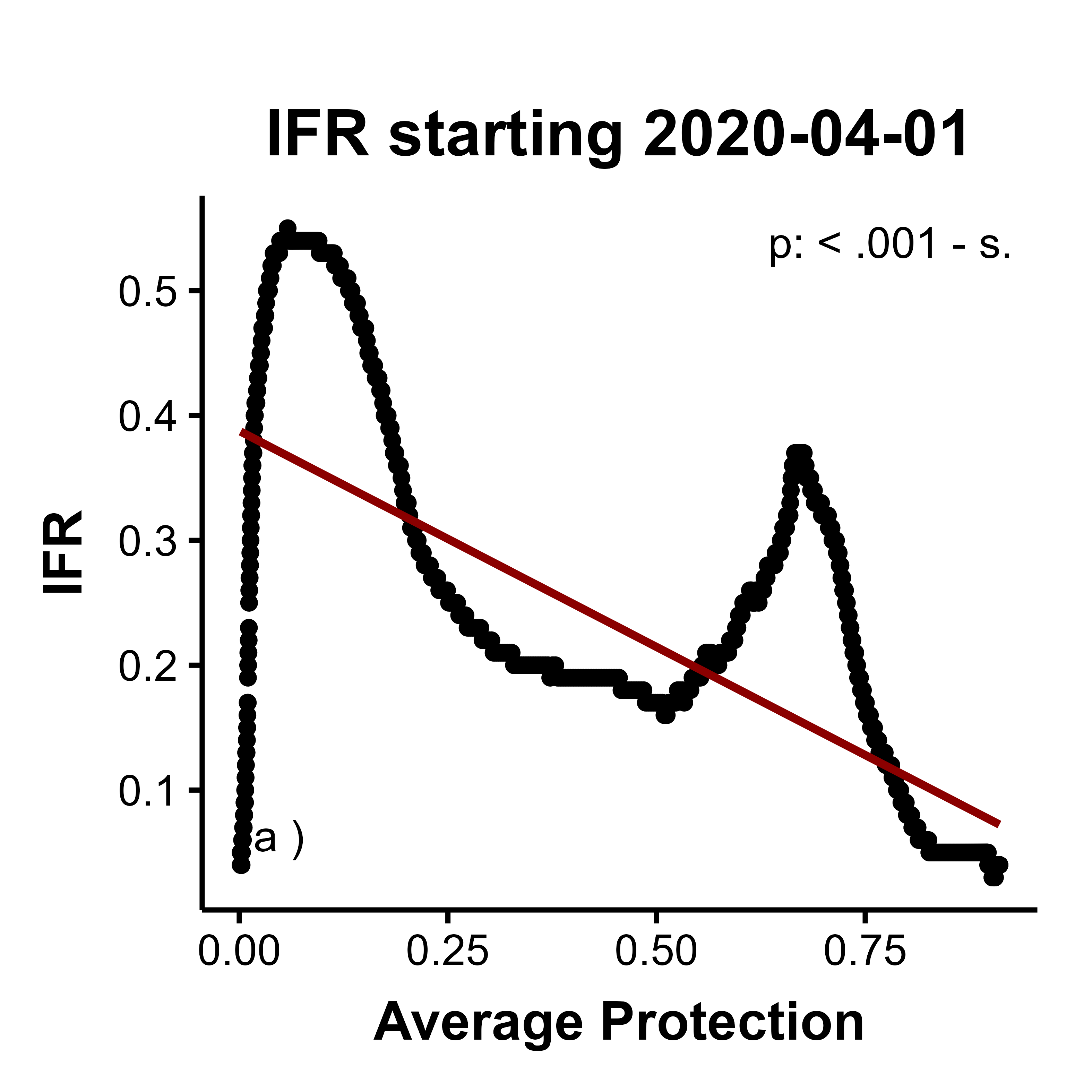

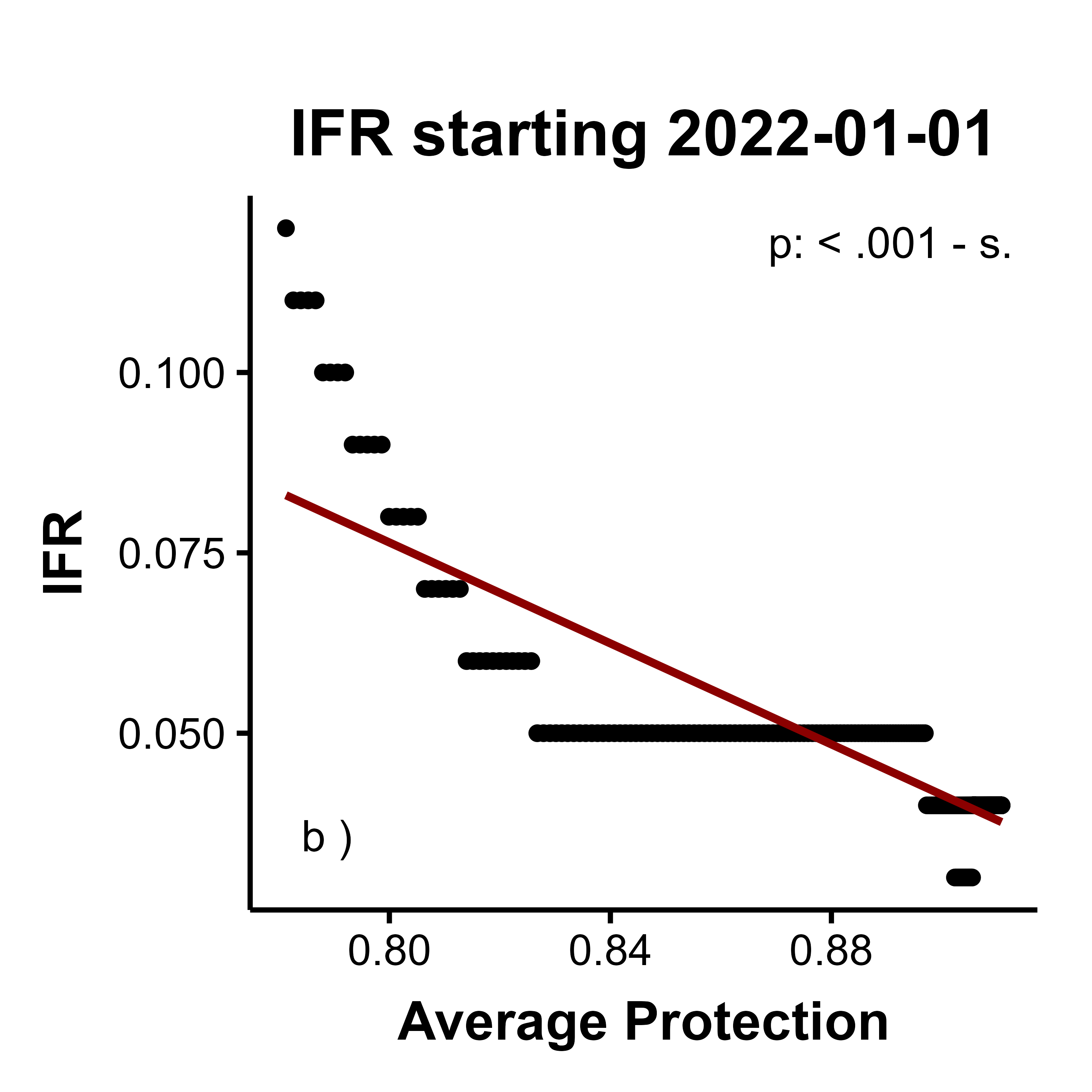


**Figure S10:** Regression lines of 4 month rolling window IFR on 4 month rolling window DP.

## **Sensitivity Analyses**

| **Table S3: Different outcome values based on parameter choice** | | | | | | | | | | | | | |
| --- | --- | --- | --- | --- | --- | --- | --- | --- | --- | --- | --- | --- | --- |
|  | **Total infected** |  | **DP (95% CI)** |  |  | **IP (95% CI)** |  | **At least one immune conferring event (95% CI)** | |  | **At least one infection (95% CI)** | | |
| **Parameter** | **Overall** | **After May 23** | **May 5, 23** | **May 23, 24** | **Amount retained (%)** | **May 5, 23** | **May 23, 24** | **May 15, 22** | **March 15, 23** | **May 23, 24** | **May 15, 22** | **March 15, 23** | **May 23, 24** |
| **Main Analysis** | 17 771 418 | 3 226 417 | 89.86% (89.86% - 89.87%) | 82.37% (82.36% - 82.37%) | 91.66% (91.65% - 91.66%) | 37.41% (37.40% - 37.41%) | 22.67% (22.67% - 22.67%) | 96.25% (96.25% - 96.26%) | 98.78% (98.78% - 98.79%) | 99.31% (99.31% - 99.32%) | 75.98% (75.96% - 75.99%) | 91.17% (91.15% - 91.18%) | 94.97% (94.95% - 94.98%) |
| **Varied infected** |  |  |  |  |  |  |  |  |  |  |  |  |  |
| **overall:** |  |  |  |  |  |  |  |  |  |  |  |  |  |
| **75%** | 13 328 564 | 2 419 813 | 85.85% (85.82% - 85.88%) | 77.46% (77.43% - 77.50%) | 90.23% (90.20% - 90.26%) | 31.29% (31.26% - 31.31%) | 18.04% (18.03% - 18.05%) | 92.98% (92.91% - 93.04%) | 96.65% (96.62% - 96.68%) | 97.75% (97.72% - 97.78%) | 62.04% (61.97% - 62.11%) | 80.48% (80.40% - 80.56%) | 86.79% (86.71% - 86.87%) |
| **90%** | 15 994 276 | 2 903 775 | 88.55% (88.52% - 88.58%) | 80.69% (80.66% - 80.72%) | 91.13% (91.10% - 91.15%) | 35.14% (35.12% - 35.17%) | 20.88% (20.87% - 20.90%) | 95.11% (95.05% - 95.17%) | 98.14% (98.11% - 98.18%) | 98.87% (98.85% - 98.90%) | 70.85% (70.76% - 70.93%) | 87.68% (87.60% - 87.76%) | 92.45% (92.38% - 92.52%) |
| **110%** | 19 548 560 | 3 549 059 | 90.90% (90.88% - 90.92%) | 83.74% (83.72% - 83.76%) | 92.13% (92.11% - 92.14%) | 39.40% (39.37% - 39.43%) | 24.35% (24.34% - 24.37%) | 97.21% (97.17% - 97.25%) | 99.23% (99.21% - 99.26%) | 99.60% (99.58% - 99.61%) | 80.48% (80.37% - 80.60%) | 93.81% (93.77% - 93.85%) | 96.75% (96.71% - 96.79%) |
| **125%** | 22 214 272 | 4 033 021 | 92.06% (92.04% - 92.07%) | 85.42% (85.40% - 85.44%) | 92.79% (92.77% - 92.81%) | 42.10% (42.08% - 42.13%) | 26.74% (26.72% - 26.75%) | 98.28% (98.25% - 98.31%) | 99.65% (99.64% - 99.66%) | 99.84% (99.83% - 99.85%) | 86.14% (86.08% - 86.19%) | 96.58% (96.53% - 96.62%) | 98.41% (98.38% - 98.45%) |
| **after pandemic** |  |  |  |  |  |  |  |  |  |  |  |  |  |
| **75%** | 16 964 814 | 2 419 813 | 89.87% (89.84% - 89.89%) | 80.66% (80.64% - 80.68%) | 89.76% (89.73% - 89.78%) | 37.38% (37.37% - 37.40%) | 19.01% (19.00% - 19.02%) | 96.26% (96.22% - 96.31%) | 98.79% (98.76% - 98.82%) | 99.21% (99.19% - 99.23%) | 76.02% (75.95% - 76.10%) | 91.19% (91.14% - 91.24%) | 94.22% (94.18% - 94.26%) |
| **90%** | 17 448 776 | 2 903 775 | 89.87% (89.84% - 89.89%) | 81.69% (81.67% - 81.71%) | 90.90% (90.88% - 90.92%) | 37.38% (37.37% - 37.40%) | 21.21% (21.20% - 21.22%) | 96.26% (96.22% - 96.31%) | 98.79% (98.76% - 98.82%) | 99.27% (99.25% - 99.29%) | 76.02% (75.95% - 76.10%) | 91.19% (91.14% - 91.24%) | 94.69% (94.64% - 94.73%) |
| **110%** | 18 094 060 | 3 549 059 | 89.87% (89.84% - 89.89%) | 83.01% (83.00% - 83.03%) | 92.38% (92.35% - 92.40%) | 37.38% (37.37% - 37.40%) | 24.09% (24.07% - 24.10%) | 96.26% (96.22% - 96.31%) | 98.79% (98.76% - 98.82%) | 99.35% (99.33% - 99.37%) | 76.02% (75.95% - 76.10%) | 91.19% (91.14% - 91.24%) | 95.27% (95.23% - 95.32%) |
| **125%** | 18 578 022 | 4 033 021 | 89.87% (89.84% - 89.89%) | 83.98% (83.96% - 84.00%) | 93.45% (93.42% - 93.47%) | 37.38% (37.37% - 37.40%) | 26.17% (26.15% - 26.18%) | 96.26% (96.22% - 96.31%) | 98.79% (98.76% - 98.82%) | 99.41% (99.39% - 99.43%) | 76.02% (75.95% - 76.10%) | 91.19% (91.14% - 91.24%) | 95.70% (95.66% - 95.74%) |
| **Decreased vaccination DP*** |  |  |  |  |  |  |  |  |  |  |  |  |  |
| **50%** | 17 771 418 | 3 226 417 | 87.16% (87.13% - 87.20%) | 81.14% (81.12% - 81.17%) | 93.09% (93.06% - 93.12%) | 37.38% (37.37% - 37.40%) | 22.64% (22.63% - 22.66%) | 96.26% (96.22% - 96.31%) | 98.79% (98.76% - 98.82%) | 99.31% (99.29% - 99.33%) | 76.02% (75.95% - 76.10%) | 91.19% (91.14% - 91.24%) | 94.97% (94.92% - 95.01%) |
| **75%** | 17 771 418 | 3 226 417 | 88.51% (88.49% - 88.54%) | 81.75% (81.73% - 81.77%) | 92.36% (92.33% - 92.38%) | 37.38% (37.37% - 37.40%) | 22.64% (22.63% - 22.66%) | 96.26% (96.22% - 96.31%) | 98.79% (98.76% - 98.82%) | 99.31% (99.29% - 99.33%) | 76.02% (75.95% - 76.10%) | 91.19% (91.14% - 91.24%) | 94.97% (94.92% - 95.01%) |
| **All DPs varied** |  |  |  |  |  |  |  |  |  |  |  |  |  |
| **75%** | 17 771 418 | 3 226 417 | 67.40% (67.38% - 67.42%) | 61.77% (61.75% - 61.78%) | 91.64% (91.62% - 91.66%) | 37.38% (37.37% - 37.40%) | 22.64% (22.63% - 22.66%) | 96.26% (96.22% - 96.31%) | 98.79% (98.76% - 98.82%) | 99.31% (99.29% - 99.33%) | 76.02% (75.95% - 76.10%) | 91.19% (91.14% - 91.24%) | 94.97% (94.92% - 95.01%) |
| **90%** | 17 771 418 | 3 226 417 | 80.88% (80.86% - 80.90%) | 74.12% (74.10% - 74.14%) | 91.64% (91.62% - 91.66%) | 37.38% (37.37% - 37.40%) | 22.64% (22.63% - 22.66%) | 96.26% (96.22% - 96.31%) | 98.79% (98.76% - 98.82%) | 99.31% (99.29% - 99.33%) | 76.02% (75.95% - 76.10%) | 91.19% (91.14% - 91.24%) | 94.97% (94.92% - 95.01%) |
| **110%** | 17 771 418 | 3 226 417 | 96.01% (95.98% - 96.03%) | 88.92% (88.90% - 88.94%) | 92.62% (92.60% - 92.64%) | 37.38% (37.37% - 37.40%) | 22.64% (22.63% - 22.66%) | 96.26% (96.22% - 96.31%) | 98.79% (98.76% - 98.82%) | 99.31% (99.29% - 99.33%) | 76.02% (75.95% - 76.10%) | 91.19% (91.14% - 91.24%) | 94.97% (94.92% - 95.01%) |
| **IPs at 75%** |  |  |  |  |  |  |  |  |  |  |  |  |  |
| **infection** | 17 771 418 | 3 226 417 | 89.49% (89.46% - 89.52%) | 82.04% (82.01% - 82.06%) | 91.67% (91.65% - 91.69%) | 34.72% (34.70% - 34.75%) | 21.05% (21.03% - 21.06%) | 95.84% (95.80% - 95.89%) | 98.55% (98.53% - 98.57%) | 99.17% (99.14% - 99.19%) | 74.13% (74.05% - 74.22%) | 89.95% (89.88% - 90.03%) | 94.18% (94.12% - 94.24%) |
| **vaccinations** | 17 771 418 | 3 226 417 | 89.80% (89.77% - 89.82%) | 82.34% (82.32% - 82.36%) | 91.70% (91.68% - 91.72%) | 37.42% (37.39% - 37.44%) | 22.66% (22.65% - 22.67%) | 95.76% (95.71% - 95.81%) | 98.64% (98.61% - 98.66%) | 99.23% (99.21% - 99.25%) | 76.90% (76.84% - 76.97%) | 91.80% (91.75% - 91.86%) | 95.34% (95.29% - 95.40%) |
| **infection + hybrid** | 17 771 418 | 3 226 417 | 88.53% (88.50% - 88.56%) | 81.15% (81.13% - 81.18%) | 91.67% (91.64% - 91.70%) | 27.07% (27.06% - 27.09%) | 16.51% (16.49% - 16.52%) | 95.02% (94.97% - 95.07%) | 98.00% (97.97% - 98.04%) | 98.81% (98.79% - 98.84%) | 70.66% (70.59% - 70.73%) | 86.97% (86.88% - 87.05%) | 92.16% (92.11% - 92.21%) |
| **Alternative DPs** |  |  |  |  |  |  |  |  |  |  |  |  |  |
| **decreased waning** | 17 771 418 | 3 226 417 | 91.64% (91.61% - 91.66%) | 87.81% (87.79% - 87.83%) | 95.83% (95.81% - 95.85%) | 37.38% (37.37% - 37.40%) | 22.64% (22.63% - 22.66%) | 96.26% (96.22% - 96.31%) | 98.79% (98.76% - 98.82%) | 99.31% (99.29% - 99.33%) | 76.02% (75.95% - 76.10%) | 91.19% (91.14% - 91.24%) | 94.97% (94.92% - 95.01%) |
| **no waning** | 17 771 418 | 3 226 417 | 93.41% (93.38% - 93.44%) | 93.59% (93.56% - 93.61%) | 100.19% (100.17% - 100.21%) | 37.38% (37.37% - 37.40%) | 22.64% (22.63% - 22.66%) | 96.26% (96.22% - 96.31%) | 98.79% (98.76% - 98.82%) | 99.31% (99.29% - 99.33%) | 76.02% (75.95% - 76.10%) | 91.19% (91.14% - 91.24%) | 94.97% (94.92% - 95.01%) |
| **No Vaccination** | 17 771 418 | 3 226 417 | 82.04% (82.00% - 82.09%) | 78.02% (77.97% - 78.07%) | 95.09% (95.05% - 95.14%) | 33.22% (33.20% - 33.24%) | 21.53% (21.52% - 21.54%) | 75.11% (75.02% - 75.21%) | 90.70% (90.65% - 90.75%) | 94.63% (94.57% - 94.70%) | 75.11% (75.02% - 75.21%) | 90.70% (90.65% - 90.75%) | 94.63% (94.57% - 94.70%) |
| **Reference Values**** |  |  |  |  |  |  |  | 96.3% (95.6%- 96.9%) | 97.9% (96.4%-98.8%) | |  |  |  |
| * as a proxy for possible healthy vaccinee bias | | | | | | | | | | | | | |
| ** Seroprevalence data as reported by Siller et al. (2024)^21^ | | | | | | | | | | | | | |
| All estimates based on 15 runs; DP = death protection; IP = infection protection | | | | | | | | | | | | | |

## **Sensitivity Figures**

**
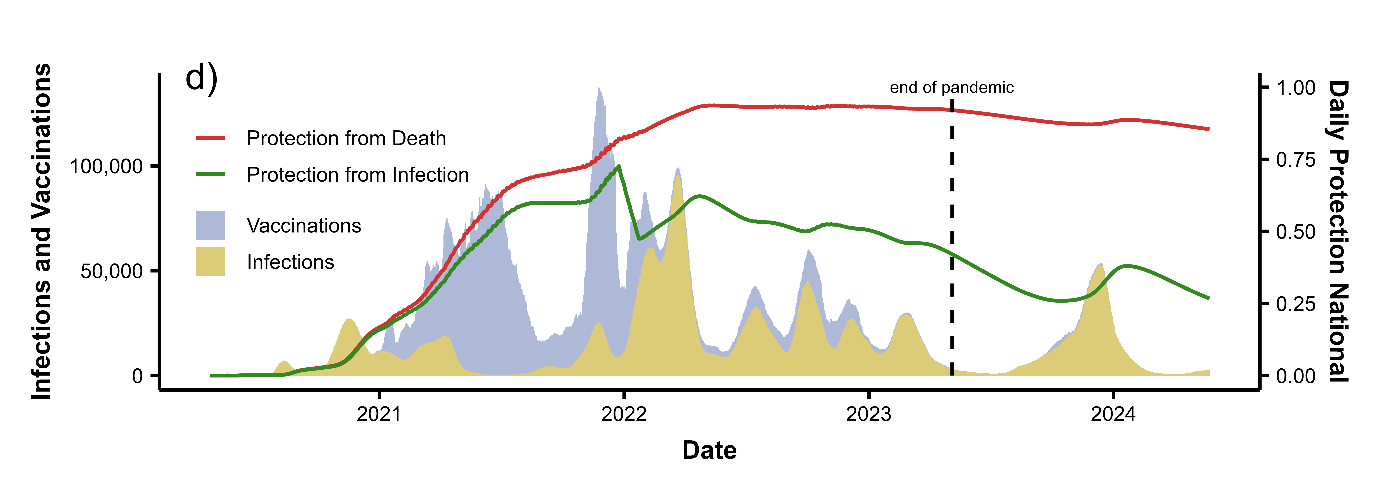
Figure S11:** Changed overall daily infections, to (a) 75%, (b) 90%, (c) 110% and (d) 125% of the original estimate.


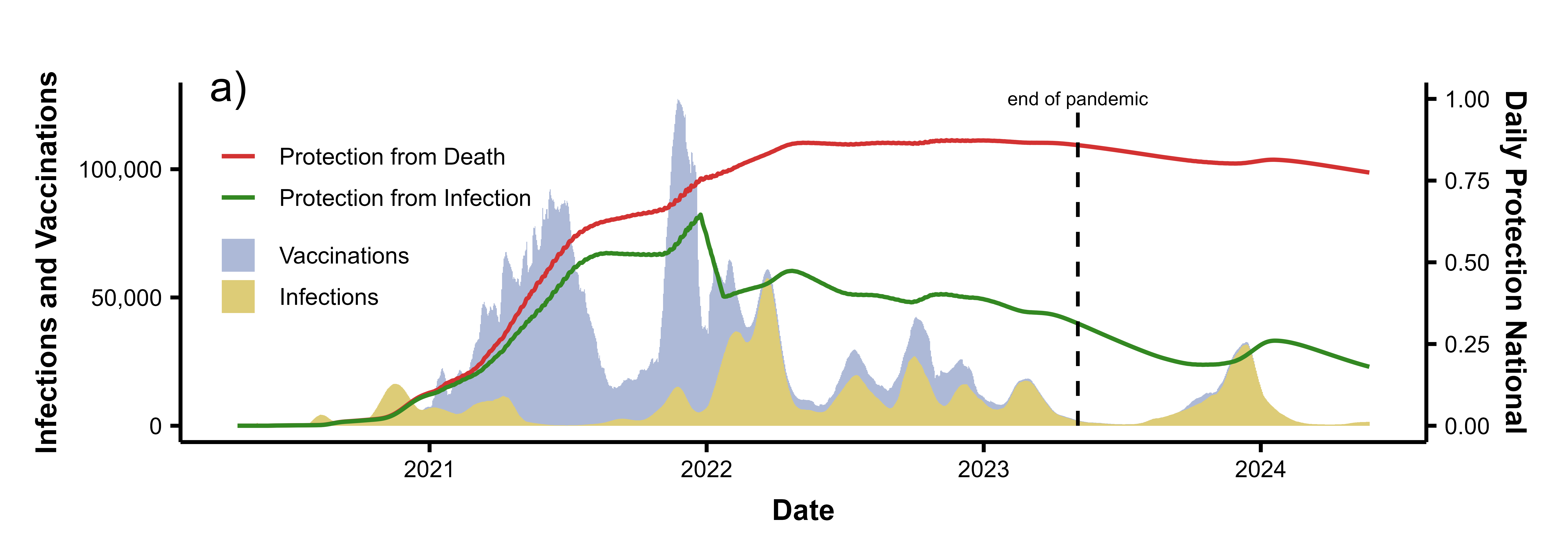

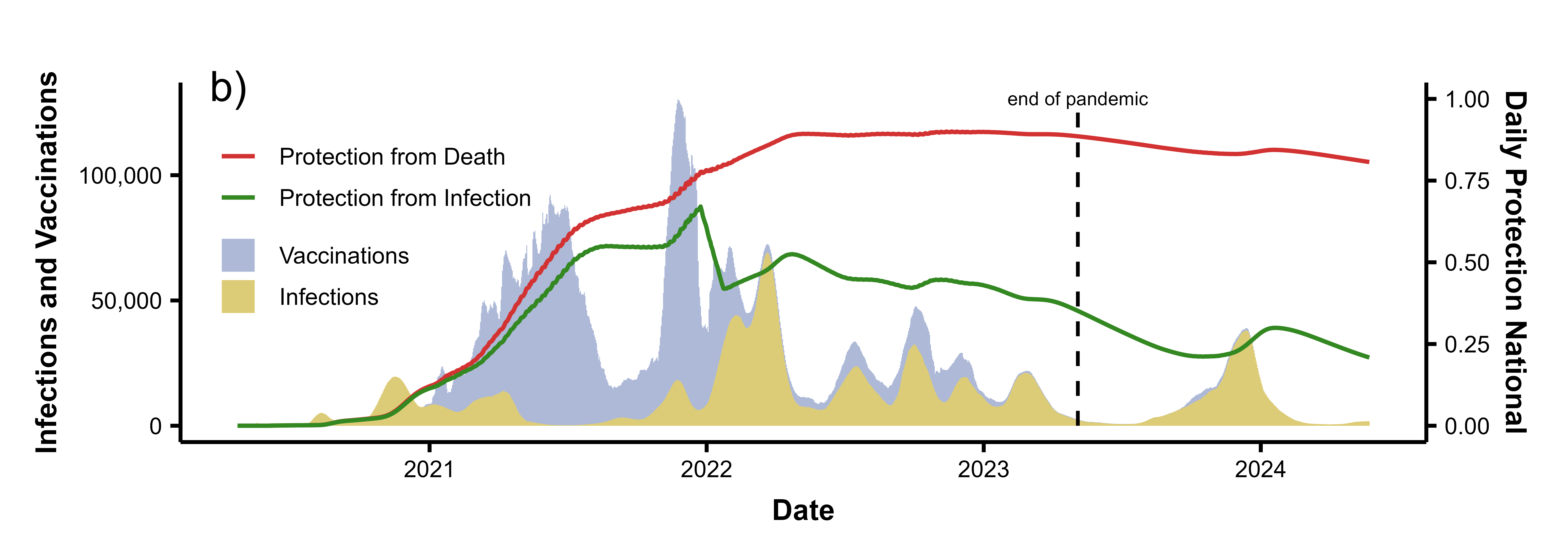

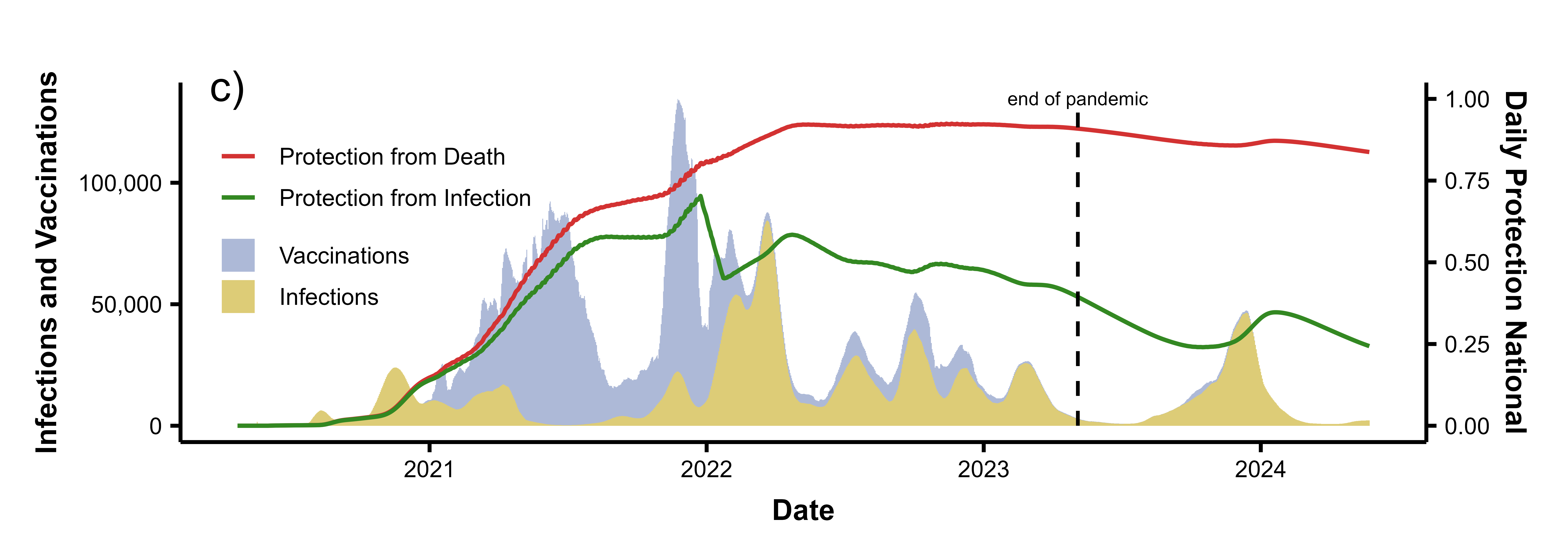


**Figure S12:** Changed daily infections after May 6, 2023, to (a) 75%, (b) 90%, (c) 110% and (d) 125% of the original estimate.


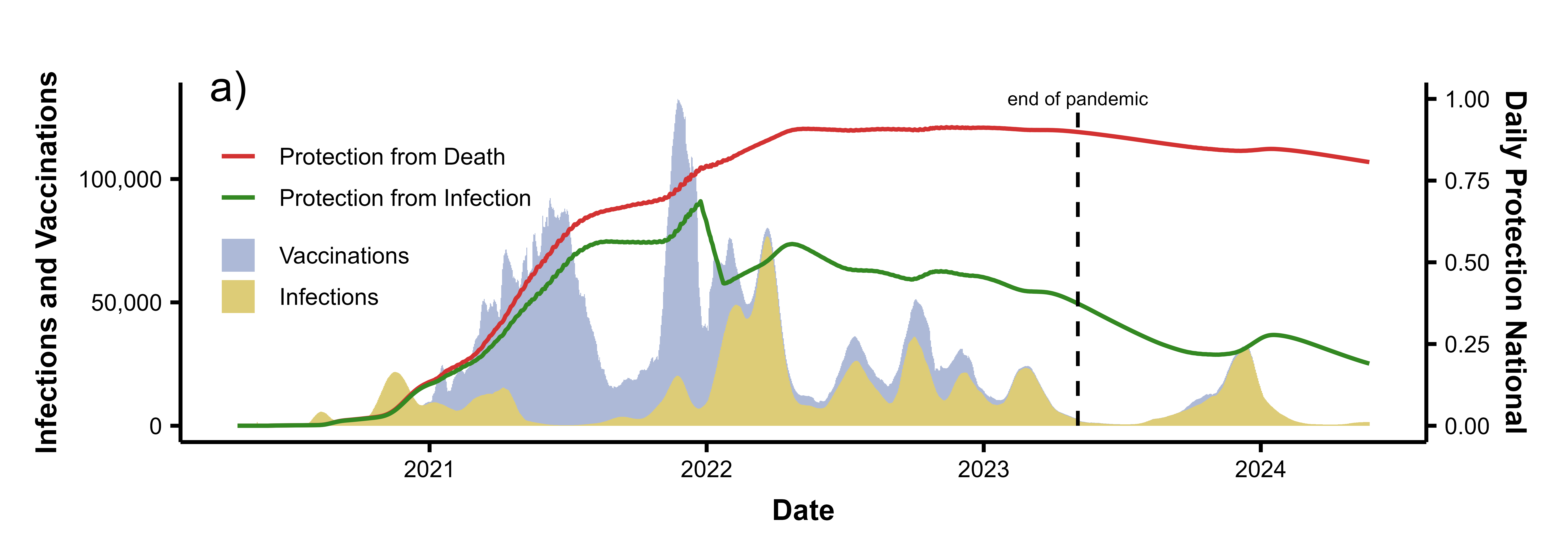

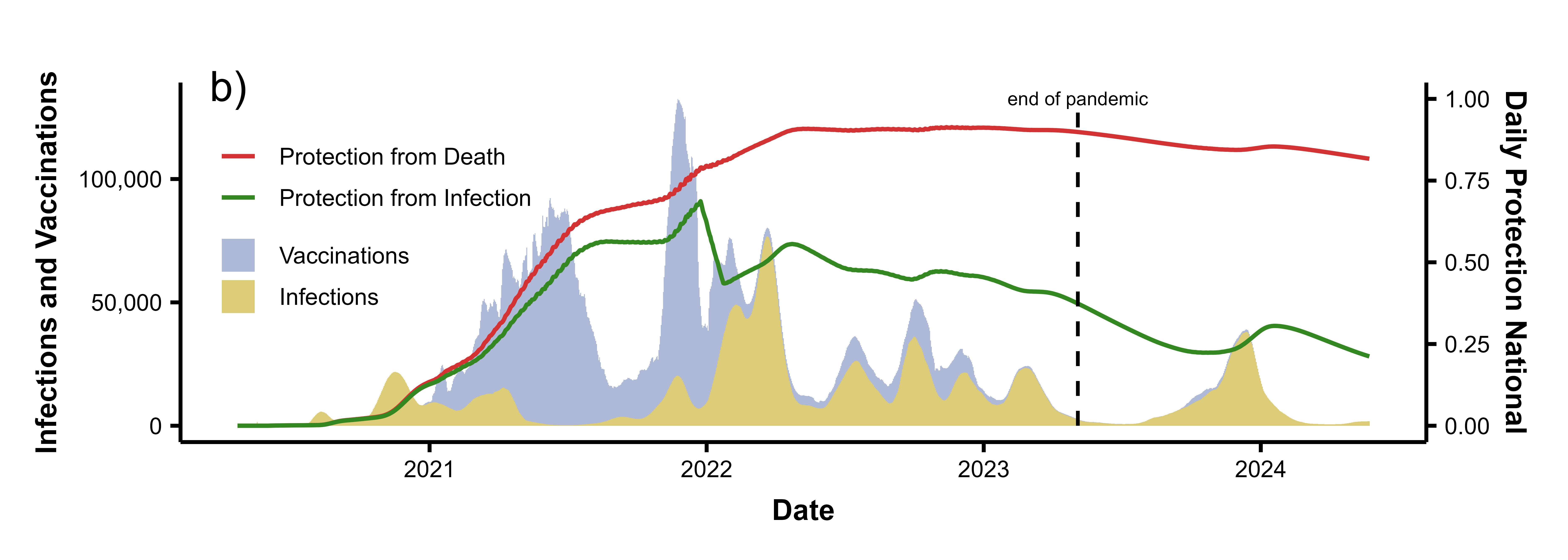

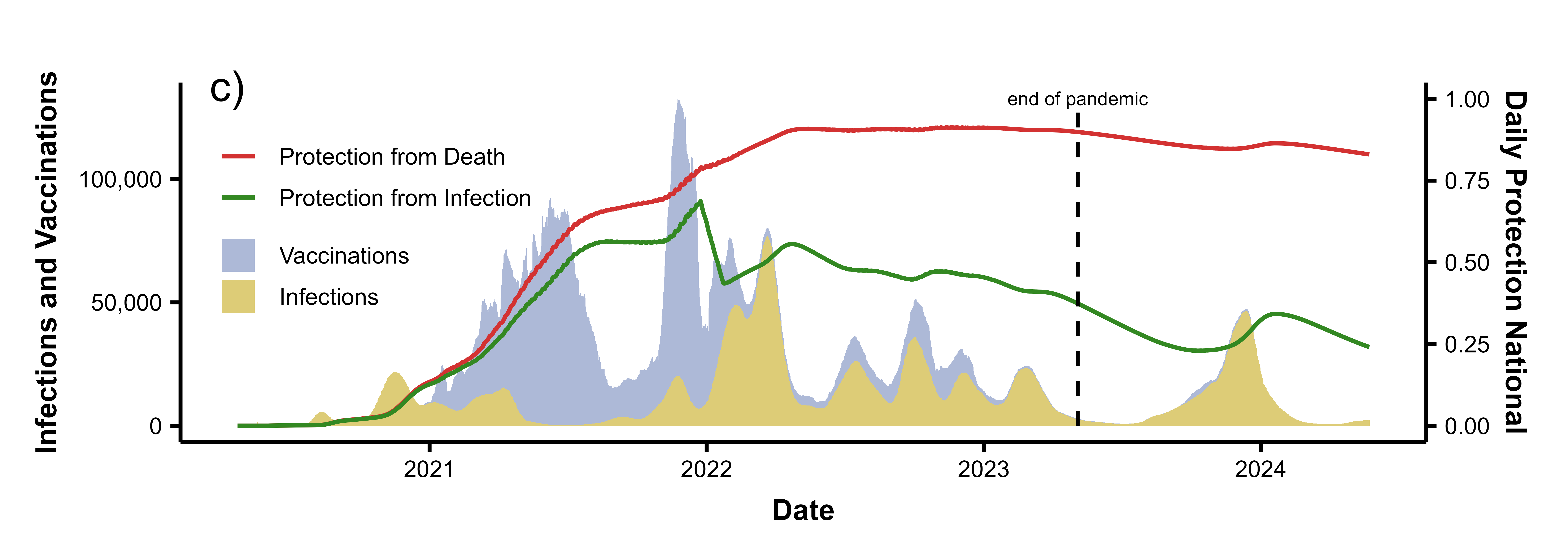

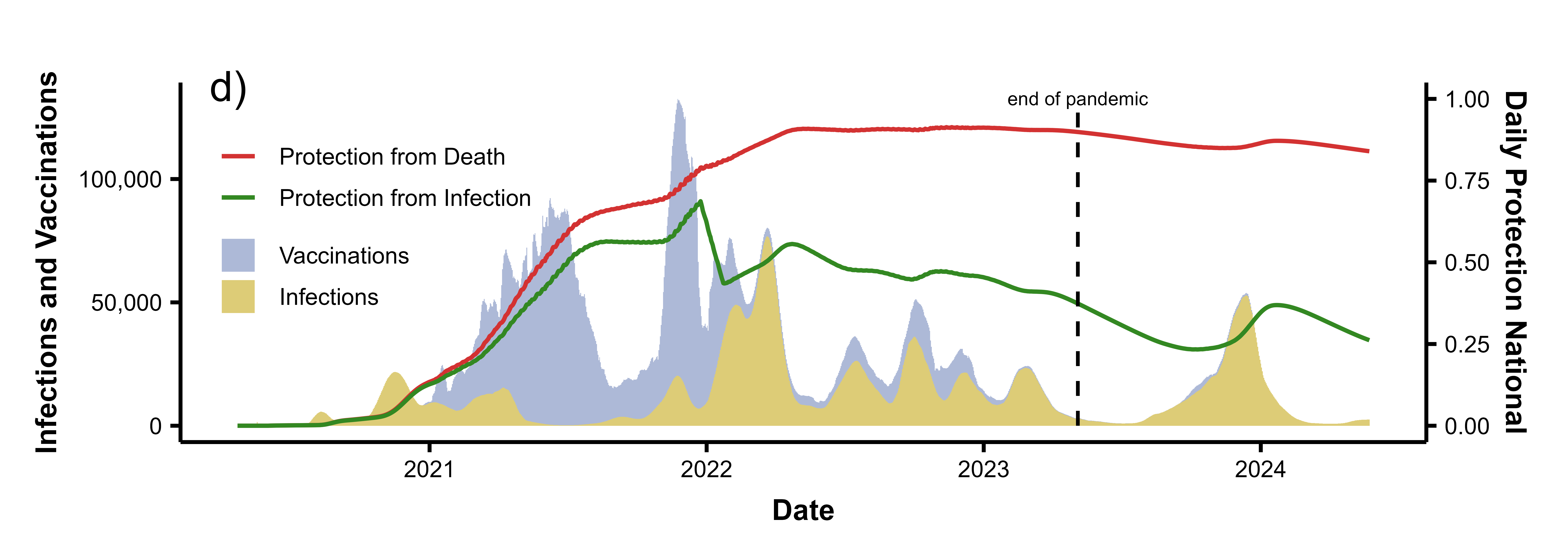


**Figure S13:** Varied all DP to (a) 75%, (b) 90% and (c) 110% of the original estimate.


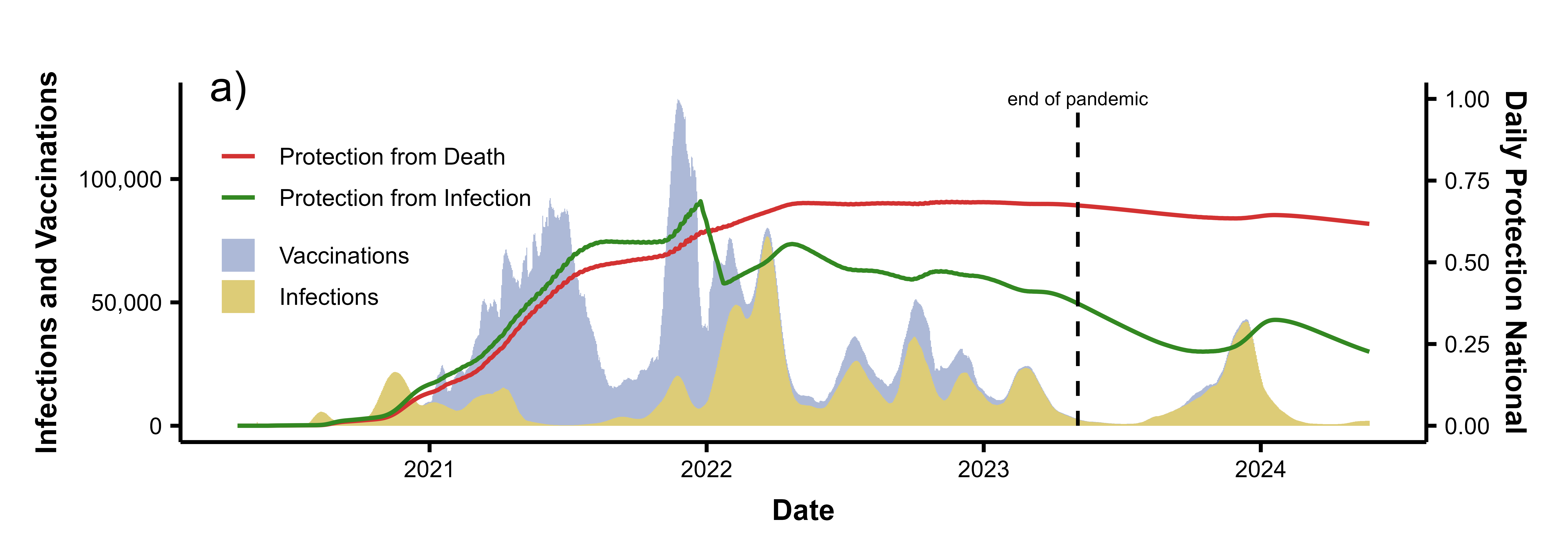

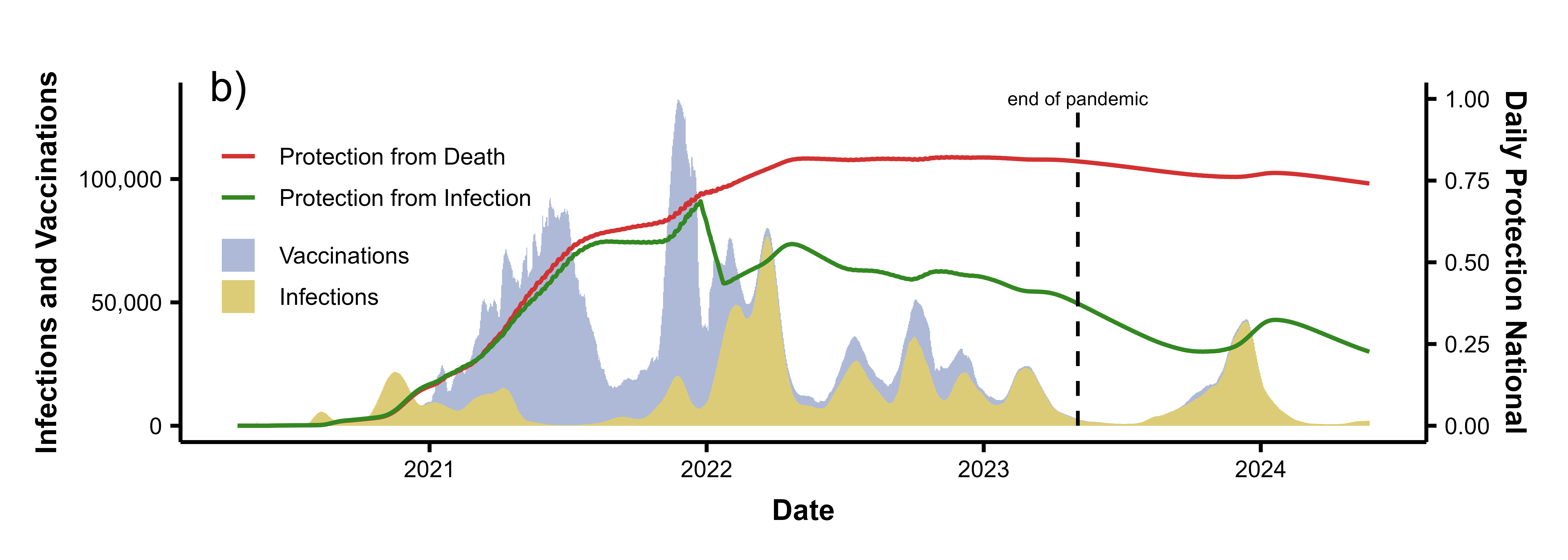

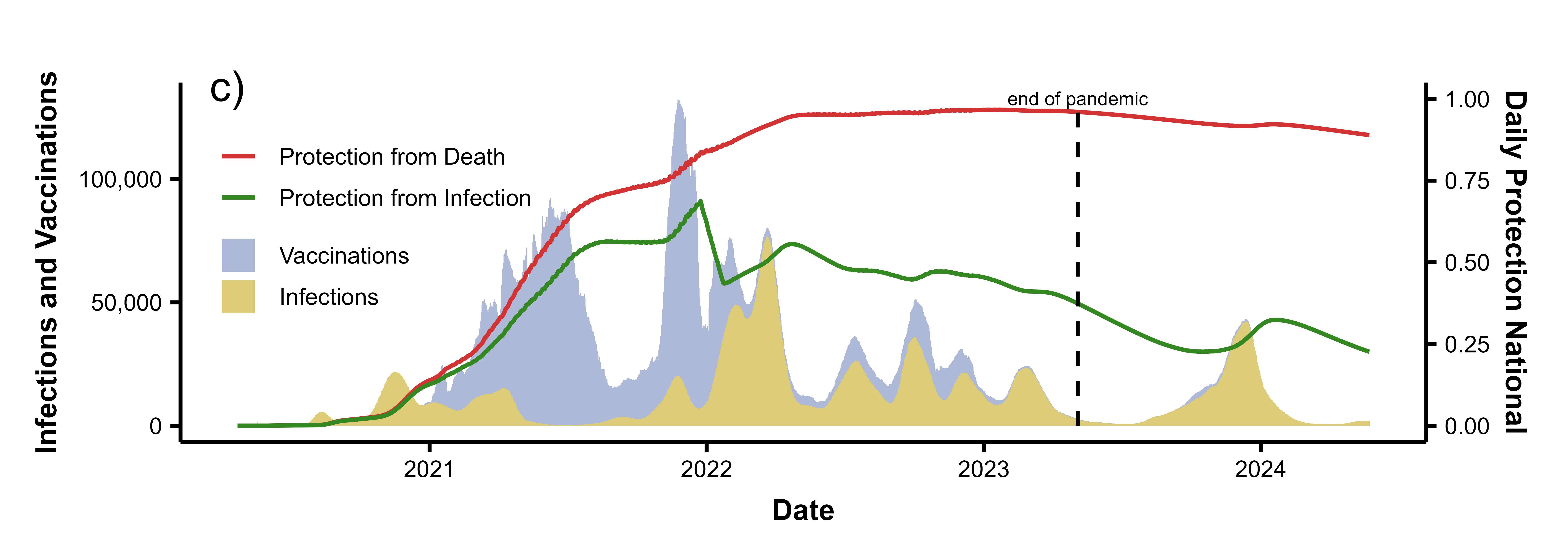


**Figure S14:** Reduced vaccination DP to (a) 75% and (b) 50% of the original estimate.


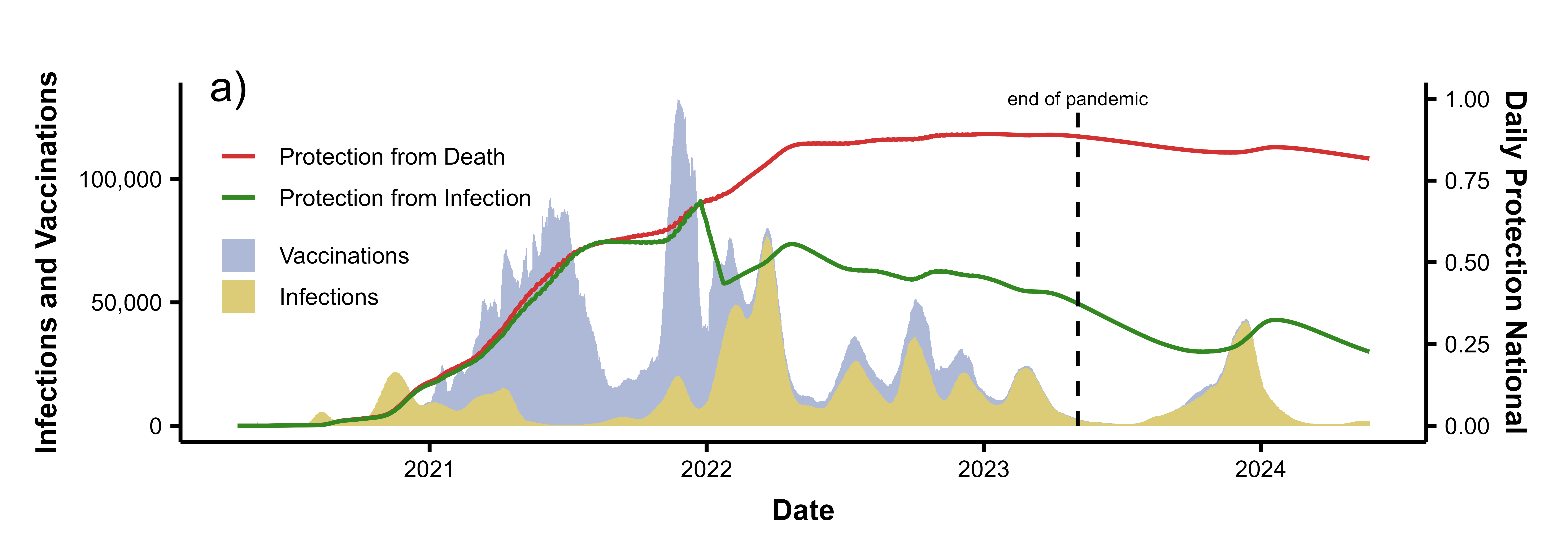

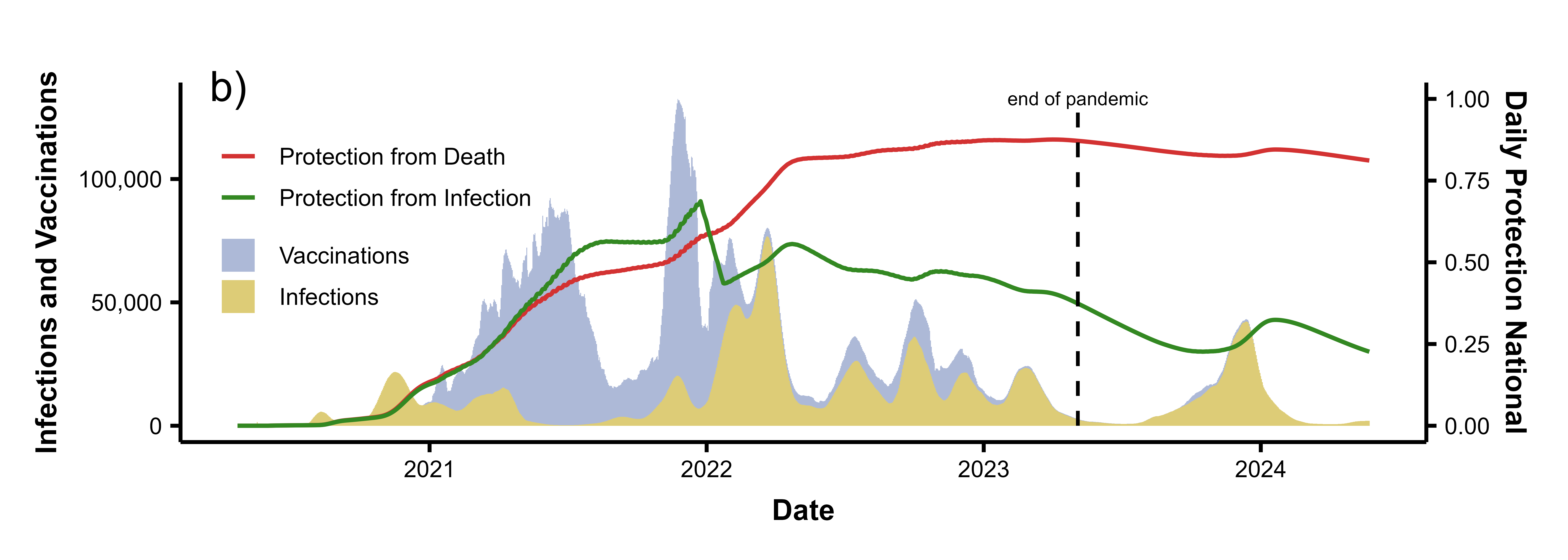


**Figure S15:** Alternative DP estimates. (a) DP mean of the main estimates and estimates without waning. (b) DP estimates without waning.


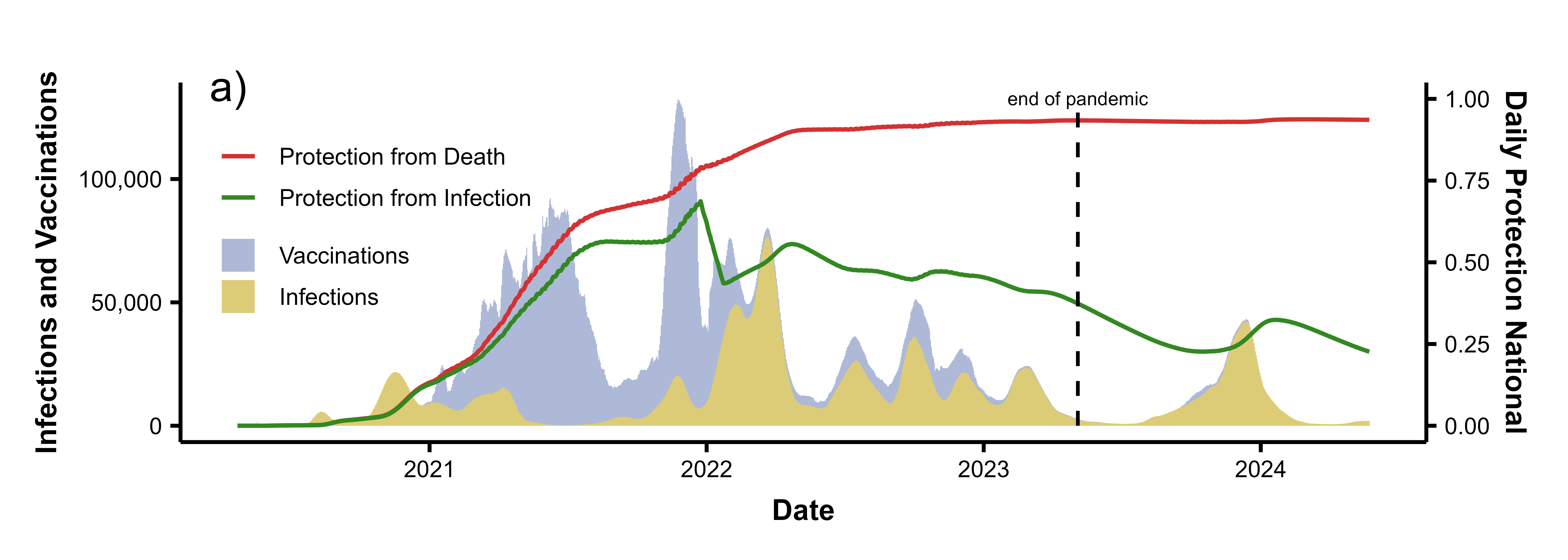

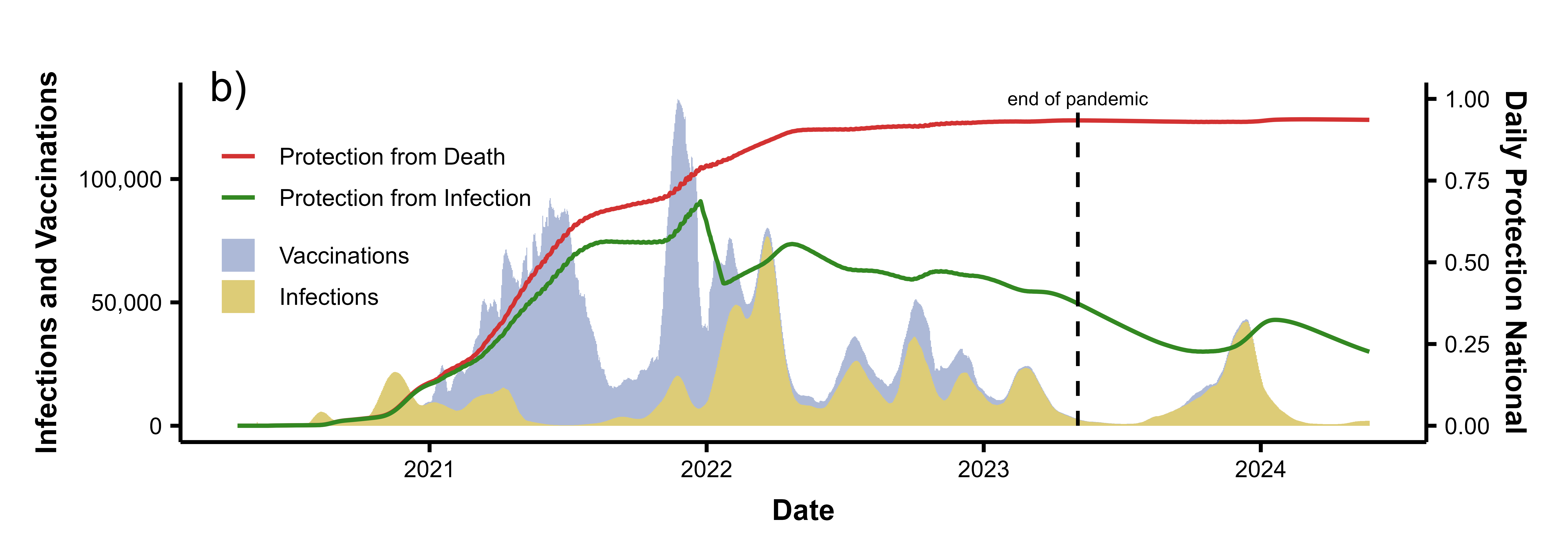


**Figure S16:** Reduced IP to 75% for different conditions. (a) IP of previous infection. (b) IP of all vaccination conditions. (c) IP of previous infection and hybrid immunity.


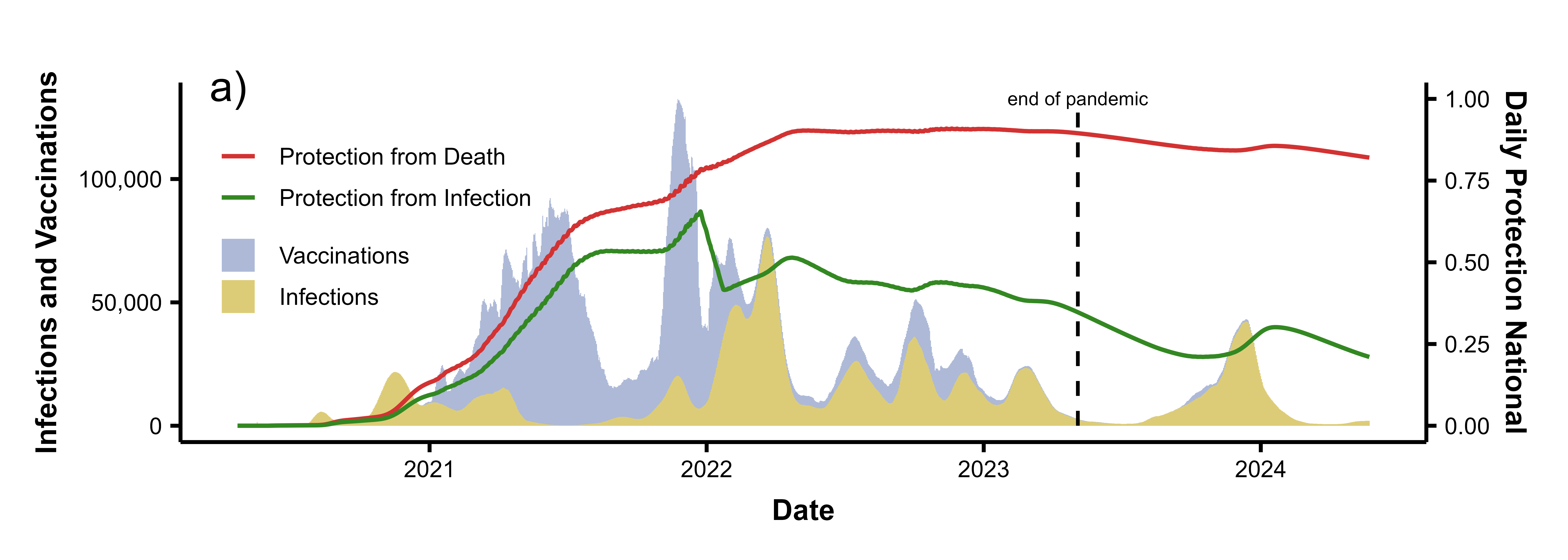

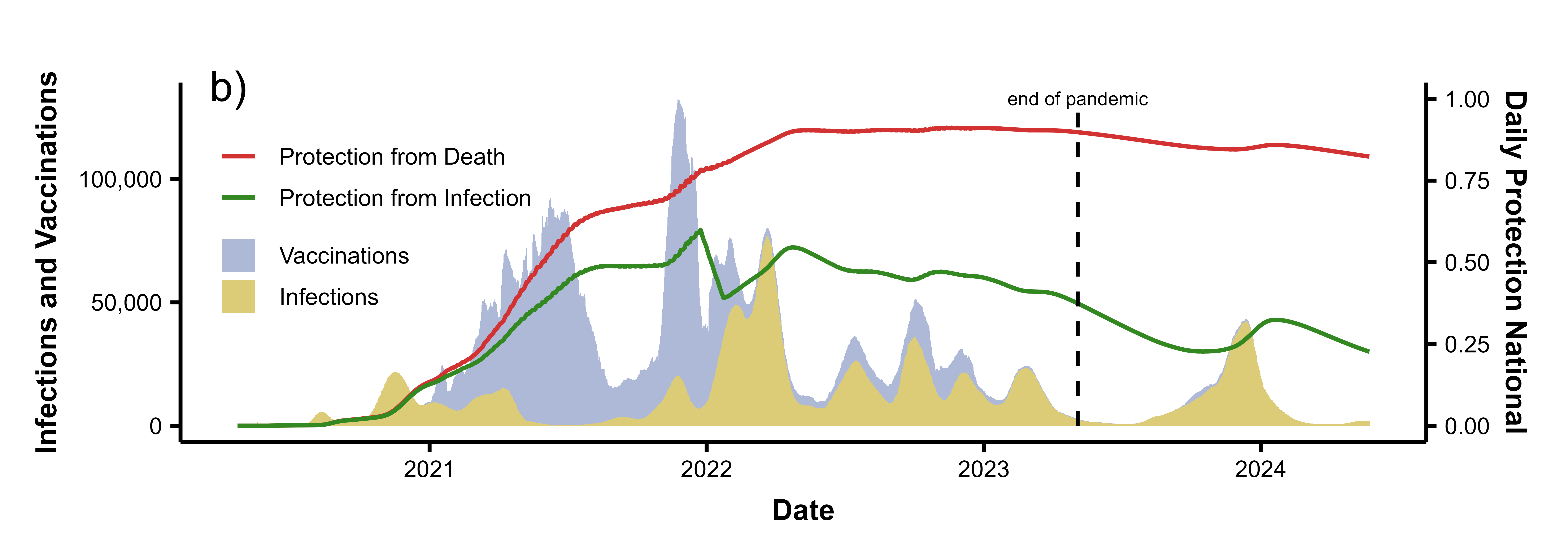

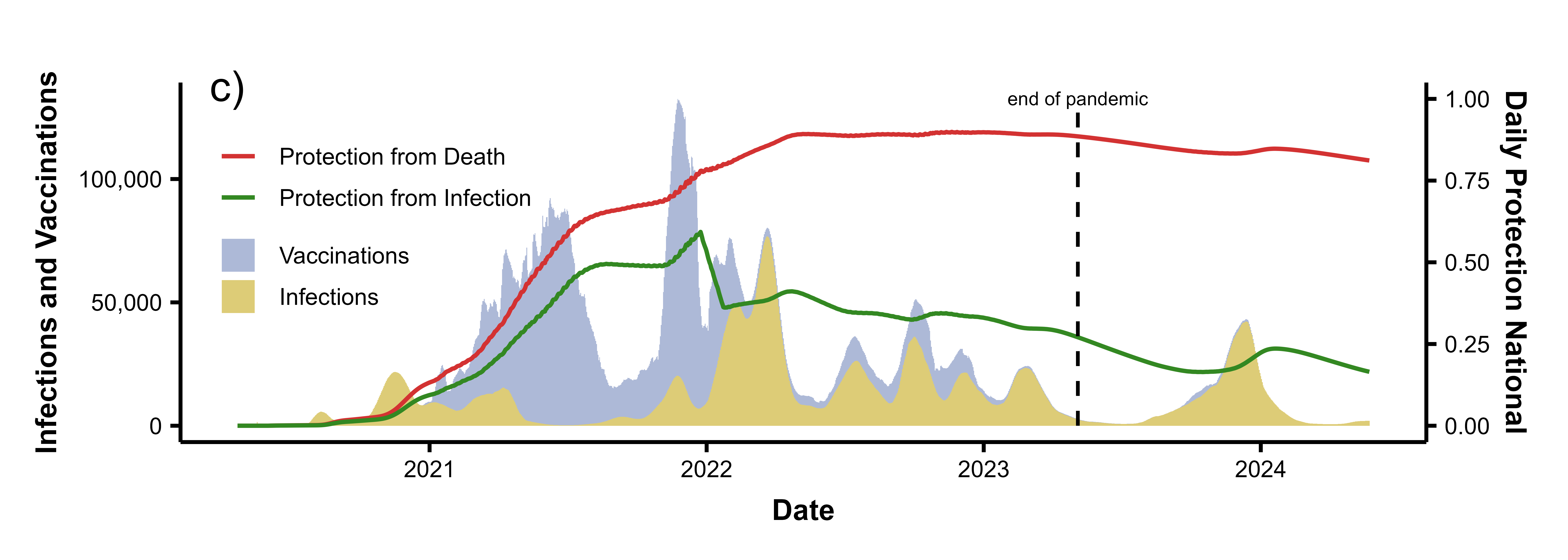


# **Supplementary Discussion**

## **Limitations**

Wastewater

There are measurement errors in the determination of the hydrochemical parameter concentration. While outliers are addressed as mentioned in the pre-processing steps, smaller errors, akin to noise, cannot be avoid.

The shedding rate varies from person to person. The average shedding rate is also not constant. The immunization rate and the virus variant are potential factors that can also influence the average shedding rate.

While we did not explicitly account for the possibility of different shedding rates of variants after the 2022, or possible effects of higher immunity on shedding rates, these possibilities are indirectly addressed by varying estimated infections for overall and post pandemic. Possible over and underestimation of up to 25% showed comparable results to the papers main findings.

Calculation of the wastewater is not based on measures from the whole Austrian population, but a smaller portion (>70% in the original estimates by Rauch^1^ and about 58% in the extrapolated estimates). This is a minor limitation, because these data are used to calculate a relative number of infected and are then scaled to the whole Austrian population. As such, wastewater plants need to be representative, which our dataset is, since the available catchment plants are located all over Austria (see Figure S17 and S18).


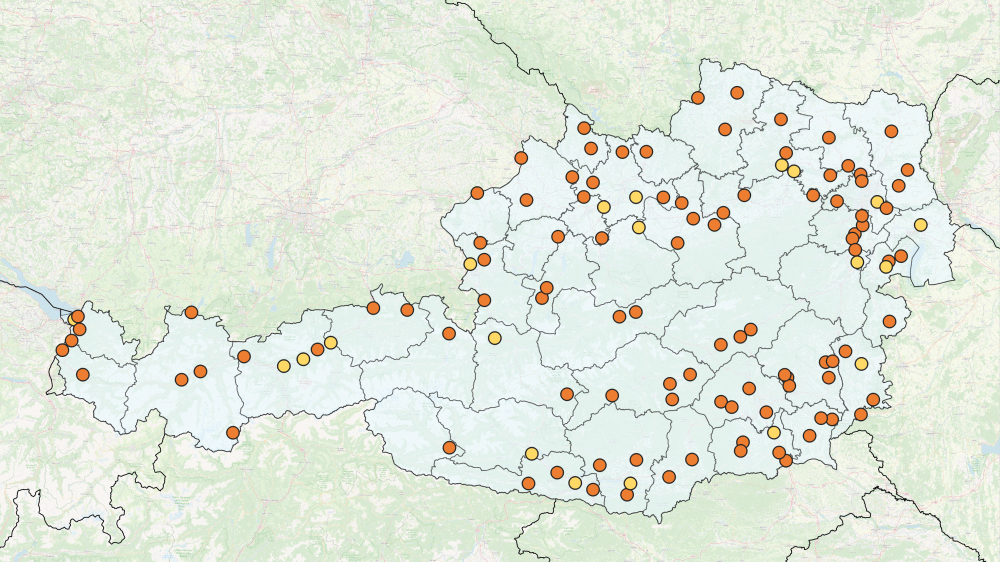


**Figure S17:** All plants from which data was included in the original investigation by Rauch et al. (2024)^1^. Their catchment areas cover over 70% of the Austrian population.


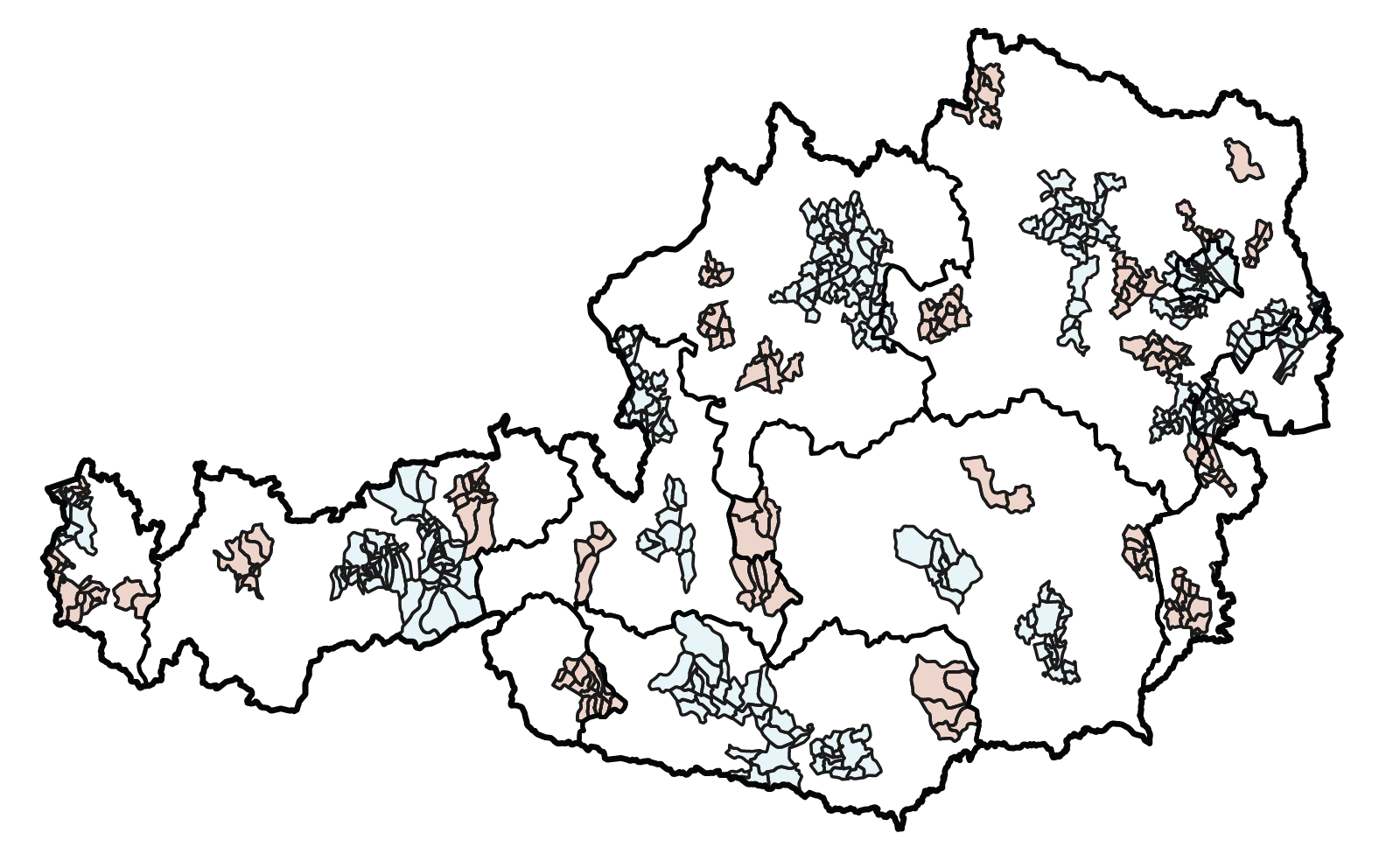


**Figure S18:** Overview of the catchment areas included in the National SARS-CoV-2 monitoring program. Approximately 58% of the Austrian population is covered.^22^ Sample collection in 24 WWTPs started in January 2022 (blue). In January 2023 another 24 WWTPs were included (red).


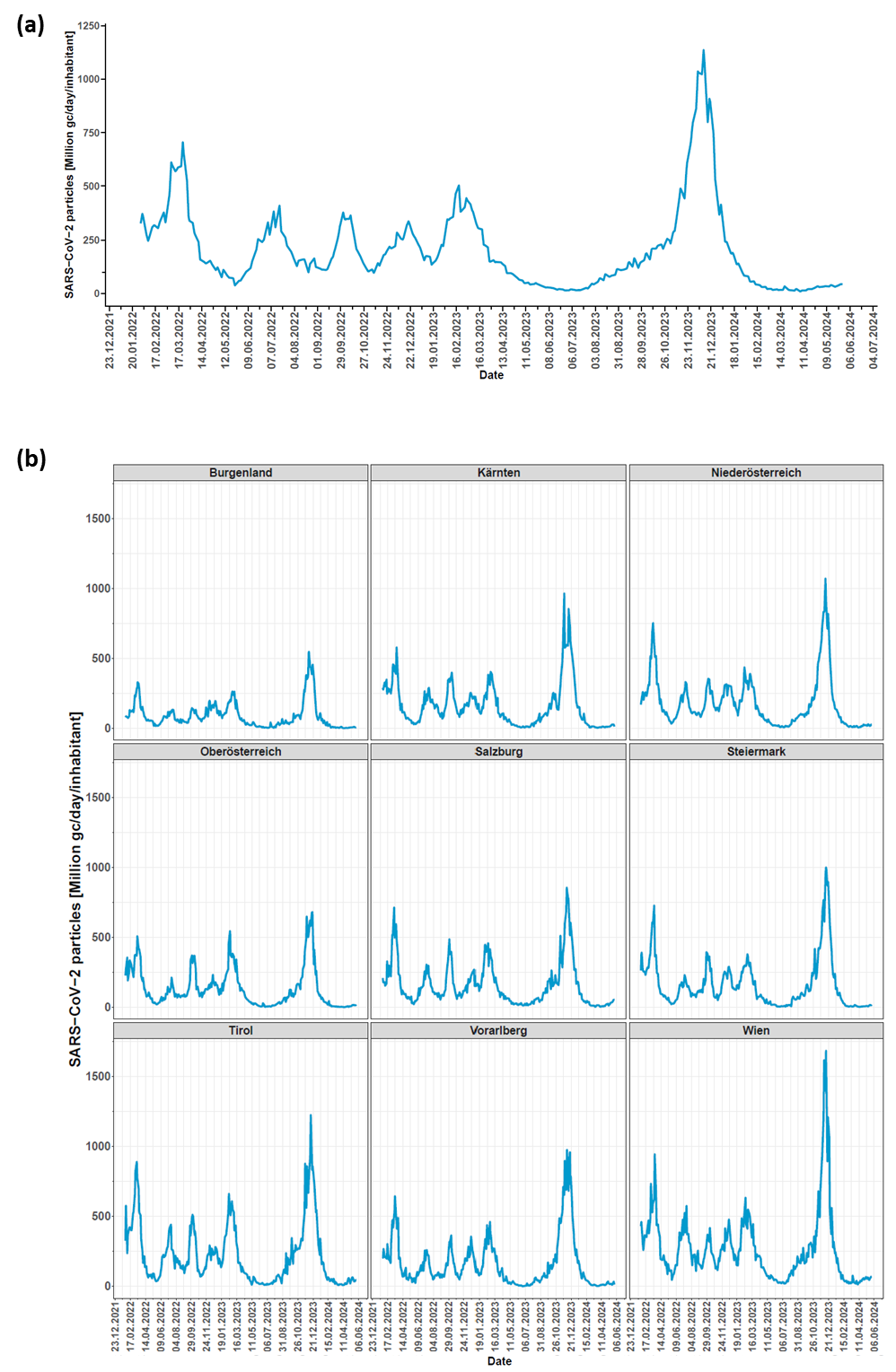


**Figure S19:** : Temporal changes of the population normalized viral loads in wastewater observed (a) at national level and (b) in the nine territories.^22^

Agent Based Model

We did not account for time between vaccinations. While there were suggestions set on time between first second and third vaccination, many early cases were rushed on purpose for high-risk groups. This could be addressed using a probability distribution based on the time since last vaccination, but we deemed the added complexity and runtime not worth the likely miniscule (if any) changes in values.

# **References**

1. Rauch W, Schenk H, Rauch N, Harders M, Oberacher H, Insam H, et al. Estimating actual SARS-CoV-2 infections from secondary data. Sci Rep. 2024;14:6732.

2. Schenk H, Heidinger P, Insam H, Kreuzinger N, Markt R, Nägele F, et al. Prediction of hospitalisations based on wastewater-based SARS-CoV-2 epidemiology. Science of The Total Environment. 2023;873:162149.

3. Arabzadeh R, Grünbacher DM, Insam H, Kreuzinger N, Markt R, Rauch W. Data filtering methods for SARS-CoV-2 wastewater surveillance. Water Science and Technology. 2021;84:1324–39.

4. Rauch W, Arabzadeh R, Grünbacher D, Insam H, Markt R, Scheffknecht C, et al. Datenbehandlung in der SARS-CoV-2-Abwasserepidemiologie. KA - Korrespondenz Abwasser. 2021;68:547–54.

5. Rauch W, Schenk H, Insam H, Markt R, Kreuzinger N. Data modelling recipes for SARS-CoV-2 wastewater-based epidemiology. Environmental Research. 2022;214:113809.

6. El Khalifi M, Britton T. Extending susceptible-infectious-recovered-susceptible epidemics to allow for gradual waning of immunity. Journal of The Royal Society Interface. 2023;20:20230042.

7. Heffernan JM, Keeling MJ. Implications of vaccination and waning immunity. Proc Biol Sci. 2009;276:2071–80.

8. Chemaitelly H, Nagelkerke N, Ayoub HH, Coyle P, Tang P, Yassine HM, et al. Duration of immune protection of SARS-CoV-2 natural infection against reinfection. J Travel Med. 2022;29:taac109.

9. Chemaitelly H, Tang P, Hasan MR, AlMukdad S, Yassine HM, Benslimane FM, et al. Waning of BNT162b2 Vaccine Protection against SARS-CoV-2 Infection in Qatar. New England Journal of Medicine. 2021;385:e83.

10. Menegale F, Manica M, Zardini A, Guzzetta G, Marziano V, d’Andrea V, et al. Evaluation of Waning of SARS-CoV-2 Vaccine–Induced Immunity: A Systematic Review and Meta-analysis. JAMA Network Open. 2023;6:e2310650.

11. Braeye T, Catteau L, Brondeel R, van Loenhout JAF, Proesmans K, Cornelissen L, et al. Vaccine effectiveness against transmission of alpha, delta and omicron SARS-COV-2-infection, Belgian contact tracing, 2021-2022. Vaccine. 2023;41:3292–300.

12. Goldberg Yair, Mandel Micha, Bar-On Yinon M., Bodenheimer Omri, Freedman Laurence S., Ash Nachman, et al. Protection and Waning of Natural and Hybrid Immunity to SARS-CoV-2. New England Journal of Medicine. 2022;386:2201–12.

13. COVID-19 Forecasting Team. Past SARS-CoV-2 infection protection against re-infection: a systematic review and meta-analysis. Lancet. 2023;401:833–42.

14. Bobrovitz N, Ware H, Ma X, Li Z, Hosseini R, Cao C, et al. Protective effectiveness of previous SARS-CoV-2 infection and hybrid immunity against the omicron variant and severe disease: a systematic review and meta-regression. The Lancet Infectious Diseases. 2023;23:556–67.

15. Rahmani K, Shavaleh R, Forouhi M, Disfani HF, Kamandi M, Oskooi RK, et al. The effectiveness of COVID-19 vaccines in reducing the incidence, hospitalization, and mortality from COVID-19: A systematic review and meta-analysis. Front Public Health [Internet]. 2022 [cited 2024 Aug 16];10. Available from: https://www.frontiersin.org/journals/public-health/articles/10.3389/fpubh.2022.873596/full

16. Wu N, Joyal-Desmarais K, Ribeiro PAB, Vieira AM, Stojanovic J, Sanuade C, et al. Long-term effectiveness of COVID-19 vaccines against infections, hospitalisations, and mortality in adults: findings from a rapid living systematic evidence synthesis and meta-analysis up to December, 2022. The Lancet Respiratory Medicine. 2023;11:439–52.

17. Grewal R, Nguyen L, Buchan SA, Wilson SE, Nasreen S, Austin PC, et al. Effectiveness of mRNA COVID-19 vaccine booster doses against Omicron severe outcomes. Nat Commun. 2023;14:1273.

18. Berec L, Šmíd M, Přibylová L, Májek O, Pavlík T, Jarkovský J, et al. Protection provided by vaccination, booster doses and previous infection against covid-19 infection, hospitalisation or death over time in Czechia. PLOS ONE. 2022;17:e0270801.

19. Stein C, Nassereldine H, Sorensen RJD, Amlag JO, Bisignano C, Byrne S, et al. Past SARS-CoV-2 infection protection against re-infection: a systematic review and meta-analysis. The Lancet. 2023;401:833–42.

20. Tomov L, Chervenkov L, Miteva DG, Batselova H, Velikova T. Applications of time series analysis in epidemiology: Literature review and our experience during COVID-19 pandemic. World J Clin Cases. 2023;11:6974–83.

21. Siller A, Seekircher L, Astl M, Tschiderer L, Wachter GA, Penz J, et al. Anti-SARS-CoV-2 IgG Seroprevalence in Tyrol, Austria, among 28,768 Blood Donors between May 2022 and March 2023. Vaccines (Basel). 2024;12:284.

22. Abwassermonitoring [Internet]. [cited 2025 Jan 9]. Available from: https://abwassermonitoring.at/
